# Contemporaneous radiations of fungi and plants linked to symbiosis

**DOI:** 10.1038/s41467-018-07849-9

**Published:** 2018-12-21

**Authors:** François Lutzoni, Michael D. Nowak, Michael E. Alfaro, Valérie Reeb, Jolanta Miadlikowska, Michael Krug, A. Elizabeth Arnold, Louise A. Lewis, David L. Swofford, David Hibbett, Khidir Hilu, Timothy Y. James, Dietmar Quandt, Susana Magallón

**Affiliations:** 1 Department of Biology, Duke University, Durham, NC 27708 USA; 2 Natural History Museum, University of Oslo, NO-0318 Oslo, Norway; 3 Department of Ecology and Evolutionary Biology, University of California, Los Angeles, CA 90095 USA; 4 Department of Biology, University of Iowa, Iowa City, IA 52242 USA; 5 Nees-Institut für Biodiversität der Pflanzen, Rheinische Friedrich-Wilhelms-Universität, 53115 Bonn, Germany; 6 School of Plant Sciences, University of Arizona, Tucson, AZ 85721 USA; 7 Department of Ecology and Evolutionary Biology, University of Arizona, Tucson, AZ 85721 USA; 8 Department of Ecology and Evolutionary Biology, University of Connecticut, Storrs, CT 06269 USA; 9 Florida Museum of Natural History, University of Florida, Gainesville, FL 32611 USA; 10 Department of Biology, Clark University, Worcester, MA 01610 USA; 11 Department of Biological Sciences, Virginia Tech, Blacksburg, VA 24061 USA; 12 Department of Ecology and Evolutionary Biology, University of Michigan, Ann Arbor, MI 48109 USA; 13 Instituto de Biología, Universidad Nacional Autónoma de México, Mexico City, 04510 Mexico

## Abstract

Interactions between fungi and plants, including parasitism, mutualism, and saprotrophy, have been invoked as key to their respective macroevolutionary success. Here we evaluate the origins of plant-fungal symbioses and saprotrophy using a time-calibrated phylogenetic framework that reveals linked and drastic shifts in diversification rates of each kingdom. Fungal colonization of land was associated with at least two origins of terrestrial green algae and preceded embryophytes (as evidenced by losses of fungal flagellum, ca. 720 Ma), likely facilitating terrestriality through endomycorrhizal and possibly endophytic symbioses. The largest radiation of fungi (Leotiomyceta), the origin of arbuscular mycorrhizae, and the diversification of extant embryophytes occurred ca. 480 Ma. This was followed by the origin of extant lichens. Saprotrophic mushrooms diversified in the Late Paleozoic as forests of seed plants started to dominate the landscape. The subsequent diversification and explosive radiation of Agaricomycetes, and eventually of ectomycorrhizal mushrooms, were associated with the evolution of Pinaceae in the Mesozoic, and establishment of angiosperm-dominated biomes in the Cretaceous.

## Abstract (web summary)

Plants and fungi interact widely and in diverse ways, from mutualism to parasitism and decomposition. Here, Lutzoni et al. analyse the timing of plant and fungal evolutionary radiations and identify four major periods in which plant-fungal interactions likely drove lineage diversification.

## Introduction

The complementarity of absorptive heterotrophy in fungi and photoautotrophy of plants has resulted in the evolution of diverse trophic interactions—both symbiotic (i.e., intimate interactions between living partners) and saprotrophic (whereby fungi drive pedogenesis and nutrient cycling via decomposition of dead plant material). Consistent with distinctive origins of such associations, these interaction types are distributed non-randomly across fungal and plant phylogenies. For example, plant pathogenic fungi (PPF) occur across much of the fungal tree of life, but reach their greatest richness and impact in the Ascomycota (especially Pezizomycotina) and Basidiomycota (especially Pucciniomycotina)^1^. The great majority (>95%) of endophytic (EF) and endolichenic fungi (ELF) are filamentous Ascomycota, concentrated in six classes of the subphylum Pezizomycotina^2^. All known lichen-forming ascomycetes (ascolichens, AL; ≈20,000 species) comprise most of the remaining classes of this subphylum^3,4^. Arbuscular mycorrhizal (AM) fungi are restricted to the Glomeromycotina, and ectomycorrhizal (ECM) fungi are primarily Agaricomycetes (Basidiomycota)^5^. In turn, every species of embryophyte and aquatic macroalgae sampled so far hosts PPF and EF^1,6,7^. The same is true for AL thalli (especially their microalgae and cyanobacteria), which host diverse ELF and lichenicolous fungi^2^. AM fungi have been reported as symbionts for 80% of extant families of vascular plants (tracheophytes), and from liverworts and hornworts^5,8^. ECM interactions mostly involve conifers (especially Pinaceae) and specific groups of woody angiosperms^5^.

Macroevolutionary specialization by fungi for specific symbioses involves diverse adaptations with regard to environmental and host-related factors. Fungi interacting with photosynthetic tissues must tolerate irradiance and desiccation, whereas associations with subterranean organs involve limited exposure to light, diminished desiccation stress, elevated CO_2_, hypoxia, and diverse interactions with soilborne microbes^9^. PPF have evolved diverse mechanisms for overcoming structural and biochemical defenses of plants, as well as host surveillance^10^, and nonpathogenic symbionts such as AM fungi have developed intricate methods for suppressing plant defenses^11^. In turn, the evolution of catabolic enzymes by fungi to degrade plant material (primarily cellulose, lignin, and associated cell wall components) is intrinsic to understanding plant and fungal evolution, relevant both to symbiosis (e.g., of horizontally transmitted EF or PPF^12^) and saprotrophy^13^.

Because most fungi depend on living or dead plants as a source of carbon, it is plausible that plants enabled the origin and diversification of major fungal lineages. Extant Viridiplantae (with an origin estimated here at ca. 1330 Ma), streptophytes (ca. 1170 Ma), and chlorophytes (ca. 1240 Ma) diversified earlier than the origin of Fungi (ca. 1020 Ma). However, the earliest known embryophytes, which had poorly developed root-like organs, likely were under enormous pressure to acquire water and nutrients^14^. Positive effects on plant growth and associated improvement of tolerance or resistance to desiccation, nutrient stress, herbivory, and diseases, are hallmarks of mycorrhizal symbioses^5^. Genetic evidence revealing AM symbiotic pathway genes in the common ancestor of embryophytes^15^, and fossils showing interactions between fungi and plants in the Rhynie Chert (410 Ma, Devonian), support a tight coupling between the origins of embryophytes and mycorrhizae, and the hypothesis that the colonization of land by embryophytes was facilitated by mycorrhizal fungi^16–19^. Moreover, benefits of AM fungi might have been amplified under elevated CO_2_ conditions that prevailed during the Palaeozoic, when extant embryophytes diversified^14^. However, increased CO_2_ availability also has been suggested as the main driving force for the adaptation of embryophytes to terrestrial environments (i.e., without necessitating their association with AM fungi)^20^. Multiple transitions to land by lineages of green algae further support the possibility that embryophytes may have adapted to a terrestrial environment without fungal symbionts^21^.

Resolving these apparently contradictory hypotheses requires robust estimates of divergence times and diversification rates for both plants and fungi. This is now possible due to improved analytical methods and expanded taxon and gene sampling, which are reflected by increasingly convergent and stable divergence time estimates derived from independent studies (Supplementary Figure 1). A diversification of terrestrial fungi before embryophytes would be consistent with a fungal-mediated colonization of land by embryophytes, whereas a diversification of terrestrial fungi after embryophytes would suggest a fungal-independent origin of terrestrial embryophytes. Based on our separate inferences of divergence times within plants and fungi, and comparison of their respective shifts in rates of diversification, we have identified four major periods when either fungi or plants likely drove the diversification of their symbiotic partner, thus impacting the evolutionary history of nearly all lineages in the tree of life.

## Results and discussion

### Fungi mediated colonization of land by embryophytes

**Fig. 1:**
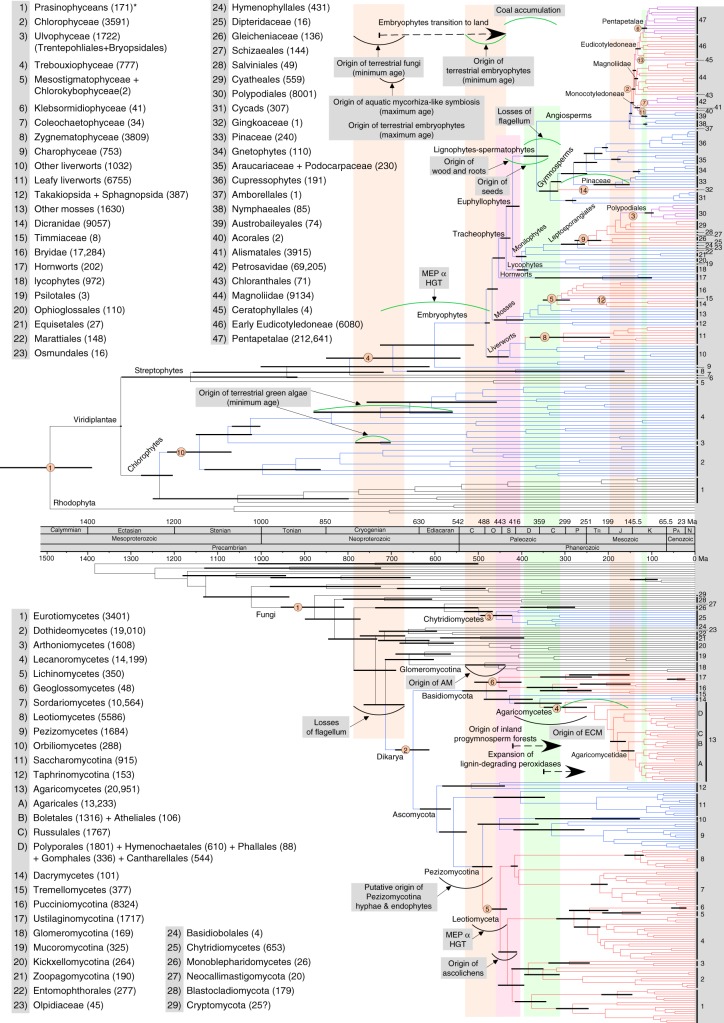
Contemporaneous changes revealed from independent estimates of divergence times for plants (top) and fungi (bottom) aligned using a single time scale. For clarity, only the most relevant error bars are shown for each divergence time estimate (see Supplementary Figs. 2, 3 for error bars). Exceptional shifts in diversification rates, highlighted by a change in color on the branches, are numbered on each tree (within yellow circles) and placed before the node where a shift was detected. The yellow circles numbered “1” indicate the basal rate relative to which the first major accelerations or decelerations were detected. Numbers at the tips of the trees refer to monophyletic groups listed to the left of each tree. Each taxon name listed on the left side is followed by its known species richness in parentheses. The asterisk after prasinophyceans indicates that this is a paraphyletic group of nine lineages of green algae currently classified at the rank of class or order, or as clades without formal taxonomic description. Three wide beige vertical bars spanning both trees highlight major events in the evolutionary history of fungi and plants that were defined by the origin of fungal key innovations, including traits that enabled mutualistic symbioses with plants. Two light green vertical bars delimit time periods when plant key innovations, such as seeds, enabled plants to form inland forests, and angiosperm diversification rates drastically accelerated, profoundly affecting the evolution of fungi. The light red vertical bar spanning both trees highlights a period when the most important diversification of plants (during the early evolution of tracheophytes) and the largest radiation of fungi (the Leotiomyceta radiation; yellow circle number 5) took place, starting around 450 Ma. Each arc represents an important event in the biology of plants (green arcs) and fungi (black arcs), including putative origins of major plant–fungal symbioses (arbuscular mycorrhizal [AM], endophytic and endolichenic [endophytes], ascolichenic [ascolichens], and ectomycorrhizal [ECM]). Horizontal dashed lines with arrows indicate major transitions from aquatic to terrestrial environments or from terrestrial wet to terrestrial dry (inland) forests, or the expansion of fungal lignin-degrading peroxidases

**Fig. 2:**
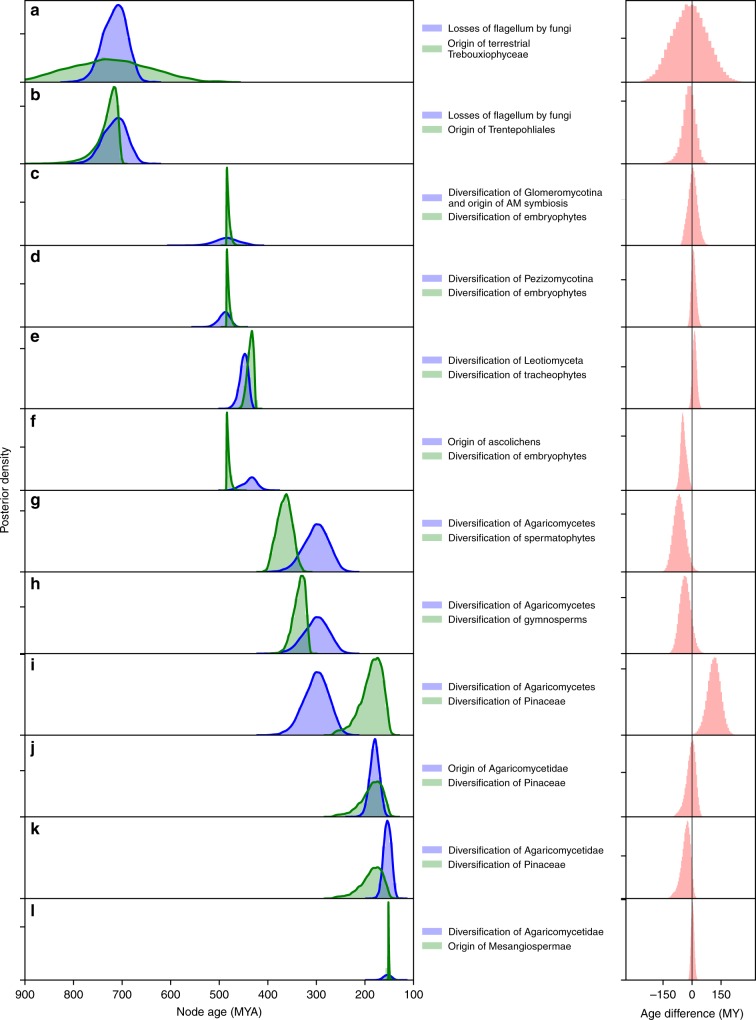
Pairwise comparison of selected land plant and fungal divergence times (node ages) posterior densities and their differences. **a**–**l** Positive age differences indicate that the fungal evolutionary event preceded the plant evolutionary event; negative age differences indicate that the plant evolutionary event preceded the fungal event

The contemporaneous origin of AM fungi and diversification of extant embryophytes reported here (Figs. 1 and 2c), as well as the earlier transition to land by fungi (ca. 720 Ma) compared to that of embryophytes, supports a fungal-mediated colonization of land by embryophytes^16^. Except for a single species (*Geosiphon pyriformis*, which is terrestrial but associated with a cyanobacterium), all extant Glomeromycotina are terrestrial, occurring as obligate symbionts of embryophytes that form AM symbioses if associated with “true” roots, or AM-like associations in non-root structures (e.g., bryophytic rhizoids). Fossil spores of Glomeromycotina have been reported as early as the Ordovician;^22^ however, no association with plants has been reported from this period. The earliest known AM fungus was reported from a fossil from the Early Devonian (≈400 Ma;^23^ Supplementary Note 1). Our analysis dates the origin of Glomeromycotina over 200 million years earlier, at ca. 659 Ma (715–606 Ma; Fig. 1, Supplementary Figure 2). During their early evolution, Glomeromycotina are thought to have associated with cyanobacteria or green algae (as exemplified by terrestrial *G. pyriformis* [Archaeosporales], an obligate mutualist of the cyanobacterium *Nostoc* that is associated with the first divergence within Glomeromycotina; Supplementary Figure 2) before partnering symbiotically with early embryophytes^19^. This is in agreement with our conservative estimates of the minimum ages for the origins of extant terrestrial chlorophytes, estimated at 725.62 Ma (784.32–702.57; divergence of *Trentepohlia* and *Tydemania*) and 726.56 Ma (881.04–561.51; crown age of terrestrial Trebouxiophyceae). Interestingly, losses of the fungal flagellum are associated with the origin of terrestrial green algae (Figs. 1 and 2a–c).

The onset of Glomeromycotina diversification is estimated at ca. 484 (529–437) Ma, providing a putative age for the origin of AM symbiosis (Fig. 1, Supplementary Table 1, Supplementary Figure 2). This timeframe is concordant with the interval between the oldest fossils that can be related reliably to embryophytes (Supplementary Figure 1; latest Middle Ordovician; Dapingian, 472–468 Ma; Supplementary Note 2), and the oldest members of the embryophyte crown group^24^ (Late Ordovician, Katian to Hirnantian, 455–444 Ma, Supplementary Note 2). The estimated date for crown embryophytes is 482 (485–473.28) Ma, reinforcing the view that AM fungi were associated with the initial radiation of plants on land (Fig. 1, Supplementary Table 2, Supplementary Figure 3). The diversification of the most species-rich subphylum of fungi (Pezizomycotina) is also contemporaneous with the diversification of embryophytes (Figs. 1 and 2d).

Some Mucoromycotina (clade 11, Fig. 1) form symbioses with early embryophytes (i.e., *Endogone*-like symbioses with Haplomitriopsida, Jungermanniopsida, Marchantiopsida, Anthocerotophytina, and ferns), suggesting that they, rather than Glomeromycotina, may have enabled plants’ transition to land^25^. In agreement with a recent genome-scale phylogenetic study^26^, we inferred Mucoromycotina as sister to Glomeromycotina (G–M clade, when excluding Mortierellomycotina, which is also part of the Mucoromycota^26^), but the divergence time of extant *Endogone* (centered around 406 Ma) appears younger than the Glomeromycotina diversification (centered around 484 Ma; Fig. 1, Supplementary Figure 2). Because the posterior density for our estimate of the origin of embryophytes is quite broad, there is a small probability that the terrestrial colonization by embryophytes could have occurred as early as 727 Ma (Fig. 1). This is within the posterior density for the origin of mycorrhizae-forming fungi (maximum age), which could have occurred during the evolution of the stem lineage of the G–M clade or the stem lineages of the Glomeromycotina–Mortierellomycotina clade and Mucoromycotina (Fig. 1).

Some Mucoromycotina (i.e., some Endogonales), as well as members of the Chytridiomycetes (Chytridiales) and Monoblepharidomycetes (Monoblepharidales) associate with early aquatic vascular plants (e.g., with *Isoëtes*; aquatic Lycopodiophyta^27^). Chytridiomycetes and Monoblepharidomycetes (C–M) diverged from their most recent common ancestor ca. 498 Ma, followed by a drastic increase in the diversification rate during the early evolution of the Chytridiomycetes (Fig. 1; minimum age estimate for diversification, 458 Ma; Supplementary Figure 2). Together, these two divergence time points (Glomeromycotina diversification ca. 484 [529–437] Ma; C–M split ca. 498 Ma), and the only drastic shift in rates of diversification that we detected outside Dikarya (i.e., Chytridiomycetes), fall within the range of minimum age estimates for the origin of terrestrial embryophytes, and for the diversification of the green algal order Sphaeropleales (many of which are terrestrial) (Fig. 1). Sphaeropleales includes *Bracteacoccus*, a genus known to associate with chytrids (Fig. 1). PPF are relatively rare in these early-arising fungal lineages, but their origins are generally consistent with the origin of their host plants. For example, *Synchytrium*, a member of the Chytridiomycetes with a wide host range, arose after the transition of plants to land, and *Rhizopus* (Mucoromycotina) arose after the origin of angiosperms (Fig. 1, Supplementary Figure 2).

When fungi first occupied terrestrial environments and began to diversify between ca. 790 and 670 Ma (based on estimated losses of flagellum by fungi; Fig. 1), they may have existed in symbiotic association with existing microbial communities, including bacteria, fungi (mycoparasitism), amoebae, and terrestrial green algae (726 Ma) (Kickxellomycotina–Zoopagomycotina clade [667 Ma] and Entomophthorales [between 661 and 715 Ma], Supplementary Figure 2). At that time embryophytes might not have differentiated from their most recent aquatic green algal relatives (Charophyceae), and therefore, may have not yet occupied land (Fig. 1). Therefore we use our maximum estimates for the origin of the embryophytes (727 Ma; Fig. 1) as the maximum age for the establishment of mycorrhiza-like associations within an aquatic or semi-aquatic environment, and as the maximum age for the occupation of land by embryophytes.

### Co-radiation of embryophytes and Leotiomyceta

The most drastic increase in diversification rates for fungi occurred during the early diversification of the Leotiomyceta, which radiated contemporaneously with the diversification of embryophytes during the Ordovician, encompassing the differentiation of tracheophytes ca. 452.32 (466.61–438.22) Ma and their initial radiation ca. 435.31 (448.53–425.73) Ma, and the diversification of Leotiomyceta around 446 (467–425) Ma (Figs. 1 and 2e; Supplementary Figs. 2-3). The co-occurrence of these events for the most species-rich subsets of the plant and fungal kingdoms is consistent with a previous report^18^, but our time estimate for this dual plant–fungal radiation is ca. 200 million years more recent and is more consistent with the fossil record (Supplementary Figure 1).

The period flanking the Ordovician–Silurian boundary represents an extraordinary phase in plant evolution, marked by the onset of substantial lineage origination and morphological diversification^28^ (particularly in terms of vegetative innovations during the following 50 My; Fig. 1). The life cycle of tracheophytes is dominated by the sporophyte, a diploid, spore-producing phase that potentially reflects enhanced capabilities for withstanding desiccation in terrestrial environments (Supplementary Note 2). By the Middle Devonian (ca. 395 Ma), terrestrial plant communities had shifted from small non-vascular assemblages to multistratified and phylogenetically diverse forests in which plants were fundamental players in structural organization, diversity, and energy flow^29^. A narrow equatorial belt characterized by warm and wet conditions associated with lowland swamps was dominated by arborescent lycophytes, which formed the tallest canopies, as well as marattialean tree ferns and sphenophytes. Contemporaneous environments, including inland areas and higher paleolatitudes, hosted different assemblages, including the incipient and subsequently diverse representation of progymnosperms (lignophytes) and their evolutionary descendants in inland forests^29^.

Extensive morphological differentiation and evolution of major lineages of fungi had already taken place when tracheophytes originated (Fig. 1). However, the rapid succession of four divergences spanning about 35 million years and encompassing the early evolution of the Leotiomyceta overlaps with the origins of tracheophytes, euphyllophytes, lignophytes–spermatophytes, and ferns, and includes (a) the origin of the Leotiomycetes–Sordariomycetes clade, (b) the divergence of the Eurotiomycetes–Arthoniomycetes–Dothideomycetes from the stem lineage leading to the Lecanoromycetes–Lichinomycetes–Geoglossomycetes splits, followed by the divergences of (c) the Eurotiomycetes and (d) the Leotiomycetes (monophyletic groups 1–8, Fig. 1). Leotiomyceta includes more than half of known fungal species (nearly 57,000 of ca. 100,000 species^4^), and likely encompasses the largest fraction of all fungi (estimated overall at ca. 5.1 million species^1^). Phylogenetic relationships among classes of the Leotiomyceta have never been resolved with confidence. Our results are consistent with the view that this reflects a rapid adaptive radiation from the late Ordovician to the Silurian.

The origin and early diversification of Leotiomyceta encompassed the emergence of novel and highly successful plant–fungal symbioses (e.g., lichenic, endolichenic, endophytic, and pathogenic; Fig. 1). The origin of extant AL is estimated to have occurred between 467 and 410 Ma (Figs. 1 and 2f), which is more recent than previously estimated^18^. Recently discovered fossils that represent the oldest known lichens, with photobionts and anatomy typical of extant lichens, suggest a new minimum age for the origin of the lichen symbiosis at 415 Ma^30^. The earliest possible origin of extant lichens must have followed the origin of Pezizomycotina hyphae (590–467 Ma; Figs. 1 and 2d), contradicting a proposed Neoproterozoic or earlier origin of extant lichens and their status as pioneer colonizers of terrestrial habitats^18^. Because EF are concentrated in the Pezizomycetes and Leotiomyceta, endophytism in the Ascomycota most likely originated in the stem lineage of the Pezizomycotina (≈590–467 Ma). It is not yet clear whether these early filamentous ascomycetes occupied embryophytes as endophytes, existed as saprotrophs, or were associated with terrestrial microalgae and cyanobacteria, perhaps as a prelude to endolichenic symbioses, followed by transitions to endophytism as new lineages of land plants originated and diversified^2^. Extant EF can markedly alter plant defenses against biotic and abiotic stress, potentially modifying plant niches over evolutionary time and providing a potential basis for diversification^6^.

This tight timing between the onset of the largest diversification of plants and radiation of fungi might be related to nitrogen uptake as a requirement for plants to successfully diversify on land, and to competition for this major nutrient if its flow is not balanced between partners in AM, ascolichenic, and endophytic symbioses^31^. Two major and independent horizontal gene transfers appear to have taken place at the onset of embryophyte diversification and Leotiomyceta evolution, where the same type of ammonium transporters/ammonia permeases (AMTPs), forming two sister clades in the methylammonium permease (MEP α) clade, were transferred from hyperthermophilic, chemoautolithotrophic prokaryotes to Leotiomyceta and embryophytes^32^ (Fig. 1). The MEP α gene was retained by all embryophytes surveyed so far and preferentially by lichen-forming fungi within the Leotiomyceta^32^. Therefore, for these symbioses to be established and maintained, only one of the symbionts can have the MEP α gene type: embryophytes, for AM and endophytic symbioses; and the mycobiont, for AL symbioses. No Glomeromycotina, endophytic Leotiomyceta, green algae, or lichen photobionts are known to have MEP α genes^32^. Reciprocal exclusion of MEP α genes may be explained by the directionality of the net flow of nitrogen in obligate symbiotic associations in nitrogen limiting environments. For example, hyphae of lichen-forming fungi most often completely cover cells of their photobionts, controlling their access to environmental sources of nitrogen. Under such conditions, the net flow of nitrogen must be from the lichen-forming fungus to its algal partner, which would require the export of nitrogen from the fungus to the alga. However, lichens living in nitrogen-rich environments or lichen-forming fungi associated with nitrogen-fixing cyanobacteria do not seem to have MEP α genes^32^. For endophytic fungi, the flow of nitrogen must be from the host plant to the residing fungus.

Importantly, the early association of fungi and plants had implications for the diversification of Dikarya (i.e., Basidiomycota and Ascomycota). The divergence of the three Basidiomycota subphyla occurred after the diversification of extant embryophytes, and much more recently (481 and 452 Ma) than did the three Ascomycota subphyla (595 and 558 Ma; Fig. 1, Supplementary Figure 2). This difference suggests that contrary to Ascomycota, the early diversification of extant Basidiomycota might have been driven by early terrestrial embryophytes. Most Agaricomycotina are associated with plants as decomposers or mycorrhizae. Approximately 90% of the Pucciniomycotina are obligate plant pathogens^33^ and virtually all known Ustilaginomycotina are plant parasites^34^. However, in contrast to the Ustilaginomycotina, which mostly parasitize angiosperms, the Pucciniomycetes are pathogenic on plant species across all main lineages of embryophytes^33,34^.

Within the Ascomycota, PPF reach their greatest diversity in non-lichenized Leotiomyceta that diversified contemporaneously with angiosperms and with the origins of major conifer lineages such as Pinaceae (Fig. 1). The distribution of other PPF in the Ascomycota is consistent with major and relatively recent events in plant evolution as reconstructed here. Extant Pezizomycetes, which include diverse ECM fungi, EF, ELF, and PPF, appear to have diversified in association with their embryophyte hosts (Fig. 1, Supplementary Figure 2). Non-pathogenic members of this class frequently reproduce sexually in habitats with low organic matter, consistent with the early divergence of the Pezizomycetes as a whole in the history of Pezizomycotina^35^. In turn, PPF are rare among the earlier-diverging lineages of Ascomycota (e.g., Saccharomycotina) with the exception of primarily plant-pathogenic Taphrinomycetes (in the Taphrinomycotina, which contains three additional classes not pathogenic on plants). The origin of pathogenicity may be recent in this class, as suggested by the recent divergence of *Taphrina* (Supplementary Figure 2), and it is consistent with the diversification of the eudicotyledon angiosperms that are their primary hosts (Fig. 1).

### Early evolution of Agaricomycetes and spermatophytes

Lignophytes—a lineage within tracheophytes that includes spermatophytes and their evolutionary precursors—are estimated to have originated ca. 420.77 (436.03–404.69) Ma, between the Early Silurian and Early Devonian. Lignophytes gave rise to many lineages, including the progymnosperms, of which only spermatophytes (seed plants) remain extant^36^. Lignophyte evolution encompassed substantial morphological innovations, including the development of wood (secondary xylem derived from a bifacial cambium), specialized vascular system and roots, and a complex branching system^36^ (Fig. 1). Seeds, which confer nutrients and protection to the developing embryo, enhance dispersal capabilities, and provide a means for reproduction without water to mediate contact between sperm and egg, evolved in only one lineage of lignophytes^36^ (Supplementary Note 2) and, according to fossil evidence, had arisen by the Mid- or Late Devonian^37^. This is substantially earlier than the diversification that yielded extant lineages of spermatophytes, estimated at 365.12 (394.9–338.39) Ma, between the Middle Devonian and the Early Carboniferous (Fig. 1, Supplementary Table 2).

The combined vegetative and reproductive innovations of lignophytes, and subsequently of spermatophytes, allowed them to occupy terrestrial areas detached from swamps and other bodies of water, and to establish shady forests formed by tall woody trees with large lateral branches and deep underground roots^29^. These inland terrestrial biomes were dominated by progymnosperms and early spermatophytes, and by the Late Carboniferous onward may have included early members of extant spermatophyte lineages. Although contemporaneous with lowland swamp communities, these inland forest biomes developed in relatively dry and cool conditions, and grew on well-oxygenated soils^29^.

The origin of spermatophytes preceded that of Agaricomycetes, but the oldest bound on estimates for their stem and crown lineages were very close (436.03 and 415 Ma for their respective stem ages, and 394.9 and 351 Ma for their respective crown ages; Fig. 1, Supplementary Figs. 2-3, Supplementary Tables 1-2). The evolution of Agaricomycetes was associated with a dramatic acceleration of diversification rates (Fig. 1) and extraordinary innovations, namely mushrooms, ECM symbioses, pathogenicity on diverse plants, and capacity to digest all components of secondary plant cell walls, including lignin^13^. Our median estimates of the stem and crown ages of Agaricomycetes are 357 Ma (415–308; Early Devonian to Pennsylvanian) and 299 Ma (351–252; Mississippian to Late Permian), respectively (Fig. 1, Supplementary Figure 2, Supplementary Table 1). The diversification of the spermatophytes seems to have taken place earlier than the Agaricomycetes radiation (Fig. 2g).

The origin of Agaricomycetes was associated with key innovations in plants such as wood, true roots, seeds, and losses of the flagellum, which allowed lignophytes to colonize dry inland areas and form forests on well-oxygenated soils that were enriched in part by highly efficient agaricomycete decomposers, and where ECM fungi provided access to phosphorus and nitrogen. For example, septate (but non-clamped) hyphae in the Devonian progymnosperm *Callixylon* may represent early Agaricomycetes^38^. However, the oldest hyphae with diagnostic clamp connections occur in ferns of Mississippian swamp forests^38^, suggesting that mushroom-forming fungi also inhabited Paleozoic coastal forests of seedless tracheophytes. Diversification of Agaricomycetes associated with the spread of inland forests of lignophytes and spermatophytes might explain the striking delay in diversification by the Basidiomycota (Fig. 1) and lower number of known species for the Basidiomycota (31,515 species) compared to the Ascomycota (64,163 species^4^) even though they diverged from a most recent common dikaryotic ancestor 649 Ma (Dikarya; Fig. 1, Supplementary Figure 2).

The evolution of lignin-degrading fungal class II peroxidases (also called Auxiliary Activity 2 enzymes [AA2] in CAZybase) and other lignocellulolytic enzymes in Agaricomycetes enabled them to function as lignin decomposers and contributors to pedogenesis^13,39^. The onset of AA2s diversification was estimated between 350 and 295 Ma^13^ (Fig. 1). The diversification of AA2s during the early evolution of Agaricomycetes may have reduced the accumulation of peat (an organic precursor of coal) at the beginning of the Permian^13^. Other non-exclusive explanations for the decline in organic carbon sequestration at the end of the Permo-Carboniferous involve shifts in climate and changes in plant investment in lignin^40^. Current understanding of peat accumulation in the tropics suggests that peat formation is mainly determined by climate, namely precipitation exceeding evapotranspiration for at least 10 months per year^41^. During the Carboniferous, differential climatic conditions yielded coal of different compositions. A highly organic coal resulting mostly from chemical degradation occurred under humid conditions in which rainfall was evenly distributed, whereas coal resulting mostly from degradation by microorganisms, and with a higher mineral content, resulted from high but seasonal humidity^42^.

The botanical origins of Paleozoic coal were dominated by lignin-rich, spore-bearing tracheophytes that did not form wood, and that proliferated and generated large amounts of biomass in wet and semi-aquatic habitats^43^. Detritivore degradation of woody plants took place before the diversification of AA2s, as indicated by the substantial contribution of Cordaites (extinct spermatophytes) to peat during specific time periods (i.e., Westphalian B-C^43^). The decline of lowland swamp communities started by the middle of the Late Carboniferous (Moskovian; 312–306 Ma), slightly earlier than the dated crown diversification of Agaricomycetes and probably before the diversification of AA2s^13^, although some of these communities held over in the Far East during the Early Permian^44^. Agaricomycete mushrooms, proficient in degrading lignin, are not known to degrade wood when covered by water (i.e., in anoxic conditions or where oxygen levels are low). Therefore, the decline of coal accumulation at the end of the Permo-Carboniferous may have resulted from a shift from lowland swamp communities to inland progymnosperm forests with well-oxygenated soils dominating the landscape, where Agaricomycetes with their expanded AA2s gene family could efficiently contribute to the decomposition of dead plant biomass.

### Radiation of ECM fungi in angiosperm-dominated biomes

Angiosperms evolved numerous vegetative and reproductive innovations that allowed them to overcome abiotic stresses such as aridity, and to thrive in ephemeral or disturbed environments^45^, leading to ecological dominance relatively soon after the onset of their diversification^46^. We estimated their divergence from other spermatophytes at 365.12 (394.9–338) Ma, and their crown diversification during the Early Jurassic (151.12 Ma; 151.8–148.79 Ma), congruent with previous molecular estimates (Fig. 1, Supplementary Figs. 1, 3; Supplementary Table 2). However, the fossil record strongly indicates a younger age (140–130 Ma, Early Cretaceous; Fig. 1, Supplementary Note 2).

Our analyses detected multiple major shifts in diversification rates during the evolution of angiosperms. The earliest is associated with the evolution of Mesangiospermae, which includes the majority of angiosperm species, between 144.6 (148.07–140.57) and 141.9 (144.87–136.88) Ma^28^ (Fig. 1). The oldest Mesangiospermae fossils are approximately 125 Ma (Supplementary Note 2). The second diversification shift subtends a clade that includes the vast majority of monocotyledons (Monocotyledoneae; Fig. 1), encompassing about 20% of living angiosperms and including distinctive components of extant terrestrial biomes. The origin of this clade (Petrosavidae) is estimated between 123.93 (128.07–121.52) and 107.44 (120.59–92.85) Ma^28^. The third and largest plant diversification is associated with the evolution of Pentapetalae (Fig. 1), which includes over 70% of living angiosperm species. Extant Pentapetalae lineages, which are major components of terrestrial biomes in terms of ecological function and species richness, began to diversify between 118.67 (125.81–112.63) and 115.35 (121.93–109.66) Ma (Supplementary Table 2, Supplementary Figure 3^28^). Multiple radiations have been detected during the evolution of the angiosperms^47^. However, our taxon sampling was designed to capture 1.3 billion years of plant evolution for comparison with 1 billion years of fungal evolution, and therefore focused on symbiotic events that preceded the diversification of the Pentapetalae. Consequently, our study did not have the resolution to detect shifts in diversification rate during the last 100 million years of evolution of Viridiplantae.

Fungi that form ECM symbioses are the most diverse mycorrhizal fungi, including over 6000 species primarily in the Agaricomycetes (especially Agaricomycetidae; Fig. 1, Supplementary Figure 2). ECM fungi associate with ca. 2000 species of spermatophytes, primarily conifers in Pinaceae and certain clades of Pentapetalae (e.g., oaks, dipterocarps, and eucalypts^5^). It is conceivable that ECM symbioses arose early in the evolution of Agaricomycetes, in lineages such as Sebacinales^48^ or Cantharellales, which we estimate to have a stem age of ca. 299 Ma (Fig. 1, Supplementary Figure 2). Such ancient ECM symbioses could have involved now-extinct lineages of spermatophytes, but direct paleobotanical evidence of such associations is lacking. Moreover, the diversification of the Pinaceae and origin of the Pentapetalae occurred after the diversification of extant Agaricomycetes (Figs. 1 and 2i). Based on extant plant lineages, the minimum age for the origin of ECM symbioses could be set by the crown diversification of Pinaceae at 183.26 (233.35–149.97) Ma, close to that of the Agaricomycetidae at 154 (171–137) Ma and the origin of Mesangiospermae 144.6 (148.07–140.57) (Fig. 2k, l; Supplementary Figs. 2, 3; Supplementary Table 2). The Agaricomycetes and Pinaceae appear to have long evolutionary histories in which neither diversified extensively (i.e., a long stem phase, Fig. 1), but the Agaricomycetidae originated contemporaneously with the diversification of Pinaceae, quickly followed by the diversification of extant Agaricomycetidae (Figs. 1 and 2j, k). However, this contemporaneous origin and diversification might not be associated with the origin of the ECM symbiosis. Extant ECM fungi are highly concentrated in the Russulales, Boletales, and Agaricales (Fig. 1, Supplementary Figure 2). The diversifications of these ECM fungal lineages seem contemporaneous with the diversification of Pentapetalae (115.35 [121.93–109.66] Ma), which includes ECM-hosting plants other than Pinaceae (Fig. 1). Therefore, the origin of ECM fungi seems to have resulted from a series of plant evolutionary events including the origins of wood, seeds and roots, which enabled the origin of inland progymnospem forests with well-oxygenated soils, followed by the origin of the Pinaceae. Contrary to AM fungal symbioses, this sequence of events suggests that the origin of extant ectomycorrhizal fungi was driven by key innovations that occurred during the evolution of the lignophytes–spermatophytes lineage.

By the Late Cretaceous, angiosperms were major constituents of terrestrial ecosystems, and lineages that are important components of present-day biomes had initiated their diversification^49^. Such biomes are characterized by high species richness and a variety of growth forms, resulting in structurally complex communities with rich trophic interactions that provided ecological opportunities for renewed diversification of several independent groups. The diversification of most extant leptosporangiate ferns (Polypodiales), which we detected as being associated with a significant increase in diversification rate (Fig. 1), followed the diversification of angiosperms and resulted in the proliferation of epiphytes associated with shady and humid angiosperm-dominated forests^50^. Similarly, the main radiation of extant epiphytic lycophytes followed the establishment of angiosperm-dominated biomes^51^.

Most Agaricomycetes that are pathogenic on plants attack trees (e.g., *Armillaria*, *Phellinus*^52^) and are similar in terms of the timing of evolutionary events relative to those described above. One notable exception is *Rhizoctonia*/*Thanatephorus*, a soilborne saprotroph and facultative pathogen with a very wide host range that also occurs as a mycorrhizal symbiont in Orchidaceae^53^. More generally, the origins and diversification of most PPF in the Basidiomycota (i.e., members of the Ustilaginomycotina and Pucciniomycotina) are consistent with the origins and diversification of their major hosts^4^ (Fig. 1, Supplementary Figure 2). Ustilaginomycotina are best known as biotrophic pathogens of early-diverging angiosperms, Magnoliidae, and various eudicot and monocot clades, particularly Poales^34^. The Pucciniomycotina are best known for >7200 species of Puccinales (rusts), a diverse lineage of obligate biotrophs, some of which are heteroecious (i.e., require hosts from multiple lineages to complete their life cycles; e.g., gymnosperm and angiosperm hosts in *Cronartium* spp., monocot and eudicot hosts in *Puccinia graminis*^33^). It is therefore not surprising that these subphyla diversified in a timeframe consistent with the origin and early evolution of angiosperms and gymnosperms^34^ (Fig. 1, Supplementary Figure 2). However, most angiosperm diversification took place in association with AM fungi, which appear to have successfully colonized and facilitated the ecological success of the earliest lineages of embryophytes.

## Methods

### Fungal data assembly and analyses

We selected a six-gene fungal data set that was previously published by James et al.^3^ within the framework of the Assembling the Fungal Tree of Life project (AFToL). The data set consists of 214 taxa, 199 of which are fungi, representing five fungal phyla. Outgroups include representatives of the Metazoa, Choanoflagellida, Mycetozoa, Chromalveolata, and Viridiplantae. The six markers included in the data set are: partial 5.8S, 18S and 28S ribosomal RNA nuclear genes, elongation factor 1α (*EF1*α), and the RNA polymerase II largest and second largest subunits (*RPB1*, *RPB2*). The third positions of the three protein-coding genes in the data set (*EF1*α, *RPB1*, and *RPB2*) were removed. All microsporidian sequences were removed to avoid alignment problems and potential anomalies their extremely long branches could cause when inferring divergence time.

The program BEAST v1.7.4^54^ co-estimates phylogeny and divergence times. The analysis had a total of 9 data partitions because the first two codon positions of three of the loci were analyzed as unique partitions (the third positions were deleted, see above). Each data partition was modeled by the GTR+I+Γ substitution model. We ran three independent BEAST runs each of 100 million generations and stationarity/convergence of the three runs was visually determined using Tracer v.1.5^55^. After the removal of 10 million generations as burn-in, the three runs were combined and a summary maximum a posteriori phylogram was estimated using the BEAST utility packages LogCombiner v.1.7.4 and TreeAnnotator v.1.7.4^56^.

A total of 13 fungal fossils were used to temporally constrain the age of specific nodes in the divergence time analyses (Supplementary Note 1). Fossil ages were determined according to the oldest stratigraphic occurrence of the fossil lineage and the corresponding lower boundary age of the stratigraphic bin on the International Stratigraphic Chart ISC (International Commission on Stratigraphy 2004). For example, the minimum age given to fossils found in the Pragian Lower Devonian Rhynie Chert was 407.0 Ma (lower boundary of the Lower Devonian on the ISC) minus 2.8 Ma (margin of error as set on the ISC), for a final minimum age of 404.2 Ma. In addition to the minimum age of each node, we applied normally distributed node age priors. The combination of a normal prior distribution and a minimum age constraint is often called a truncated normal distribution. Age constraints were applied to the nodes of the fungal phylogeny from which the fossil taxonomic group diverged from its sister group. However, if a chosen clade was not supported with at least 95% posterior probability and 70% ML bootstrap proportion, the constraint was applied to the next deeper and well-supported node (see Supplementary Figure 2). While reconstructing the phylogeny, these constrained nodes were set to remain monophyletic. Nodes labeled A to M in Supplementary Figure 2 were constrained with node age priors based on fossil data discussed in Supplementary Note 1. We assigned species richness data to major lineages of the BEAST fungal chronogram using Kirk et al.^4^ (Supplementary Figure 4), and used MEDUSA^57^ to identify exceptional shifts in diversification rates with the R package Geiger^58^.

### Plant data assembly and analyses

Phylogenetic inferences among green plants are based on the combined sequences of nine plastid genes (cpLSU, cpSSU, *rps4*, *trnL*, *atpB*, *psaA*, *psbB*, *rbcL*, and *matK*), including the catalytic core of the *trn*L group I intron from a sample representing two major primary plastid lineages, green plants, and red algae. Sampling within green algae represented the two phyla Chlorophyta and Streptophyta (Supplementary Figure 3). Within Chlorophyta, we included members of early diverging lineages, collectively known as “prasinophytes” but which are multiple distinct classes, including Palmophyllophyceae (*Prasinococcus*). We also sampled within the diverse classes Chlorophyceae, Ulvophyceae, and Trebouxiophyceae (Fig. 1); all of these classes contain members that are symbiotic with fungi. Within Streptophyta, we sampled six algal classes, including Mesostigmatophyceae and Chlorokybophyceae, which together form the earliest diverging lineage in the phylum. Also included were representatives of three classes that have been placed as sister to land plants (embryophytes), i.e., Coleochaetophyceae, Zygnematophyceae, and Charophyceae. Sampling of land plant lineages was guided by Magallón et al.^28^ for seed plants and complemented with more representative sampling of early diverging land plant lineages, liverworts, mosses, and hornworts as well as lycophytes and ferns (Fig. 1, Supplementary Figure 3).

Sequences were obtained from previous studies as well as genome data present in GenBank (Supplementary Data 1). Alignment of each gene was performed using the MUSCLE algorithm^59^ as implemented via the plugin option in PhyDE-1 v0.9971^60^. Based on the criteria laid out in Kelchner^61^, incorrect motif recognition and placement were manually corrected. Areas of ambiguous alignment were excluded from the analyses (Supplementary Table 3). In the case of the *trnL* intron only the catalytic core was included, whereas the stem-loop regions P8 and P9, apart from the closing stem P8.0 and P9.0, the terminal loops of P1, P5, and P6 and the inner bulge (partly) between P4 and P5 as well as the γ-arm of the *trnL* 3′-exon (variable extra arm of various tRNAs) were considered as alignment hot spots^62^. The concatenated matrix comprised 17,709 characters (Supplementary Table 4).

Phylogenetic relationships were estimated with RAxML-HPC v.8^63,64^. Five independent analyses were performed, each simultaneously searching for the best-scoring ML tree, and conducting a rapid bootstrap analysis of 1000 iterations, implementing GTRCAT with 25 distinct substitution rate categories. PartitionFinder v1.1.1^65^ suggested the following portioning scheme, which was applied in the phylogenetic reconstructions: (cpLSU, cpSSU) (*atpB12*) (*atpB3*, *psbB3*) (*matK*) (*psaA12*) (*psaA3*) (*psbB12*) (*rbcL12*) (*rbcL3*) (*rps4*) (*trnL*). The starting tree was obtained with parsimony. The five analyses converged into congruent topologies and parameter values. We randomly chose one of the five RAxML analyses, and used the resulting ML phylogram in subsequent dating analyses.

Dating analyses used the uncorrelated lognormal relaxed clock implemented in BEAST v1.8.3^56^. The data were the same as those used in phylogeny estimation, but were partitioned into nuclear markers (6188 sites), plastid protein-coding (9853 sites), and plastid non-coding (1668 sites). Each partition was analyzed with a GTR+I+Γ model that was unlinked across partitions. A Birth–Death model was implemented as a tree-generating prior, with a user-specified ultrametric tree estimated with r8s^66^ as the starting tree. The root node of the tree, representing the divergence between red algae and green plants, was assigned a uniform distribution between 1393 and 1623 Ma, derived from the age interval estimated for this divergence in Tree E of Parfrey et al.^67^, including 109 taxa representing the main eukaryote lineages; considering Proterozoic and Phanerozoic fossils as calibrations; with *Bangiomorpha* providing a minimum age for red algae of 1174 Ma; and estimating the position of the root of the tree. Forty-one internal nodes were calibrated with the oldest fossils belonging to relevant clades, with their phylogenetic position and calibrated node assigned on the basis of morphological considerations, and when available, on explicit phylogenetic results (see below). Thirty-nine of these calibrations were implemented as lognormal prior distributions in which, following Heath^68^, *μ*, the mean of the lognormal distribution was equal to ln(expected age) − *σ*^2^/2; the expected age was 5% of the fossil age; and *σ*, the standard deviation, was 0.75. The land plant (embryophyte) crown node was calibrated with a uniform prior distribution between 485 Ma, corresponding to the oldest fossils of embryophytes^69,70^, and 443.8 Ma, corresponding to the oldest fossils of crown embryophytes^24^. The flowering plant (angiosperm) crown node was calibrated with a uniform distribution ranging from 151.8 to 132.9 Ma, corresponding to the age interval estimated for this node by Silvestro et al.^71^. Seventeen independent BEAST analyses were conducted. An evaluation of likelihood and parameter scores of independent runs with Tracer v1.6.0^55^ revealed that several runs failed to reach convergence, hence, we considered only the five analyses that achieved the highest likelihood scores. Each of these consisted of a MCMC of 100 million generations, in which one every 5000 steps was sampled. The initial 15 million steps of each MCMC were excluded as burnin (after verifying that the chain had reached a stable phase). The effective sample size of most parameters in the combined runs was >200. Trees of the five independent analyses were combined with LogCombiner and parameters were summarized on the maximum clade credibility tree with TreeAnnotator^56^. As for the fungi data set, MEDUSA^57^ was used to identify exceptional shifts in diversification rates during the evolution of Viridiplantae with the R package Geiger^58^. The tree topology and species numbers are shown in Supplementary Figure 5.

### Comparison of Viridiplantae and Fungi chronograms

To compare the evolutionary histories of Viridiplantae and Fungi required that we consider a period of more than 1 billion years. Because our focus was on plant–fungal symbioses, our taxon sampling was designed to be as complete as possible up to the last 75 million years. By excluding tens of thousands of recently evolved angiosperm species, for which data are available, we were able to use computationally intensive methods with the most realistic evolutionary models (BEAST) to estimate phylogenetic relationships and divergence times with higher statistical confidence for both kingdoms. MEDUSA uses the “terminally unresolved tree approach” to sampling discussed by FitzJohn et al.^72^, in which “the species not explicitly included as tips in the phylogeny are all known to belong to terminal unresolved clades. This tree is therefore ‘complete’ in that it includes all extant taxa but is incompletely resolved.”^72^. Our inclusion of 21 main clades for that analysis, representing all angiosperms, with total numbers of known species for all terminal clades, enabled the detection of major shifts in diversification rates throughout the evolution of Viridiplantae, including the early diversification of the angiosperms. Adding a large number of species of angiosperms to capture recent radiations within this clade would be irrelevant to our study, because all major plant–fungal symbioses originated before recent radiations of the angiosperms.

Identifying major diversification rate shifts across the tree of life is a topic of high biological interest. Nevertheless, few methods are available that explicitly fit Birth–Death (BD) models to identify diversification shifts across the branches of a phylogenetic tree. MEDUSA^57^ is a method that uses the Akaike Information Criterion (AIC) to select among BD models with constant or variable rates, and detects the number and phylogenetic placement of diversification rate shifts. Nevertheless, concerns including its ability to correctly choose among models have been expressed^73^. BAMM^74^ is a Bayesian method that does not rely on the AIC to select among diversification models. In addition to detecting diversification rate shifts among branches, it can account for rate changes through time. Importantly, MEDUSA and BAMM differ in their treatment of sampled diversity. While BAMM uses a likelihood that assumes that species from the full tree are included randomly, MEDUSA uses the likelihood of observing the number of extant species sampled in an empirical phylogeny, given the diversification parameters (D. Rabosky, pers. comm. to SM). Therefore, in the case of substantially non-randomly undersampled empirical trees, as was the case here for both phylogenies (fungi and plants), MEDUSA is more appropriate to identify diversification shifts. The distinction between the two sampling schemes and implications for likelihoods were discussed in FitzJohn et al.^72^, but their application to diversification shift methods has not been discussed in the literature.

The posterior distributions of node age differences were estimated from the MCMC-sample output of BEAST. For each node of interest, a probability density function (PDF) was obtained by Gaussian kernel density estimation using the stats.gaussian_kde function in the Python SciPy package^75^. For each comparison of a node from the plant tree and a node from the fungal tree, 100,000 paired random samples were obtained from each of the relevant PDFs. Kernel density estimation was performed on the resulting node-pair age differences. The posterior probabilities of age differences for 1, 5, 10, 20, 40, and 60 million-year ranges were then estimated by integrating the distribution of node-age differences over the relevant interval using the “integrate_box_1d” method (Supplementary Table 5). A 95% credible interval for each node-age difference was computed using the highest posterior density (HPD) method^76^.

### Code availability

Scripts and files used for extracting node ages from posterior tree samples, summarizing posterior distributions for target clades, plot figures and computing tabular summaries are deposited in the Dryad repository [10.5061/dryad.7bn4001]^77^.

## Supplementary information

Supplementary Information

Description of Additional Supplementary Files

Supplementary Data 1

## Data Availability

Files with molecular alignments used in divergence time analyses and resulting trees are available in the Dryad repository [10.5061/dryad.7bn4001]^77^. New molecular data used in this study are deposited in TreeBase [http://purl.org/phylo/treebase/phylows/study/TB2:S12607] (plants). A reporting summary for this article is available as a Supplementary Information file.
