# Supplementary material for: Contemporaneous radiations of fungi and plants linked to symbiosis
Source: Nat Commun. 2018 Dec 21;9:5451. doi: 10.1038/s41467-018-07849-9 (PMC6303338; doi:10.1038/s41467-018-07849-9)
Supplement: Supplementary file 1 — Supplementary Information [file 41467_2018_7849_MOESM1_ESM.pdf]

Supplementary Information

Contemporaneous radiations of fungi and plants linked to symbiosis

Lutzoni *et al.*

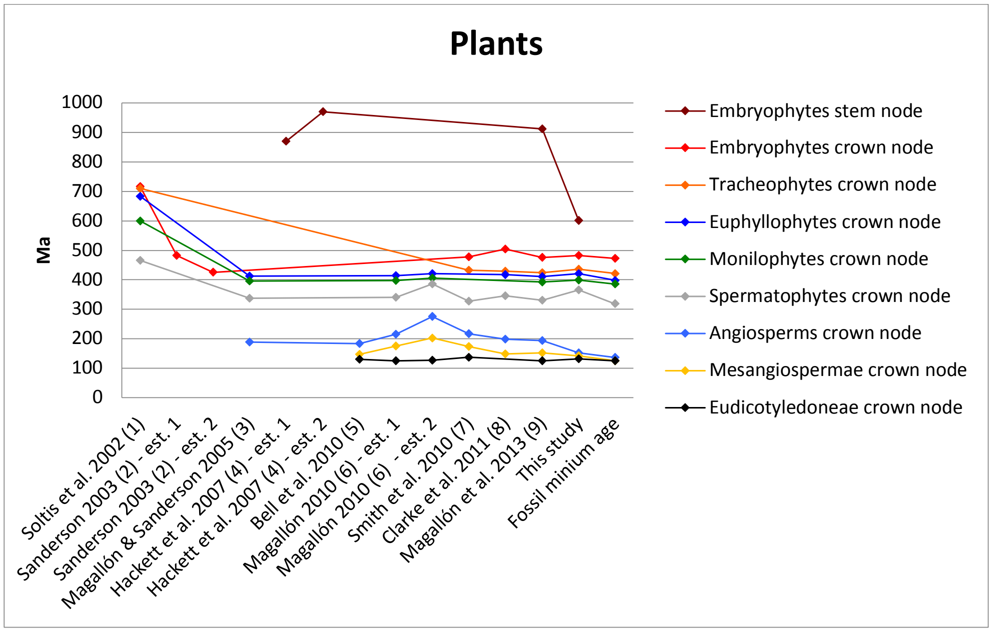
Plants
Ma
Embryophytes stem node
Embryophytes crown node
Tracheophytes crown node
Euphyllophytes crown node
Monilophytes crown node
Spermatophytes crown node
Angiosperms crown node
Mesangiospermae crown node
Eudicotyledoneae crown node
Soltis et al. 2002 (1)
Sanderson 2003 (2) - est. 1
Sanderson 2003 (2) - est. 2
Magallón & Sanderson 2005 (3)
Hackett et al. 2007 (4) - est. 1
Hackett et al. 2007 (4) - est. 2
Bell et al. 2010 (5)
Magallón 2010 (6) - est. 1
Magallón 2010 (6) - est. 2
Smith et al. 2010 (7)
Clarke et al. 2011 (8)
Magallón et al. 2013 (9)
This study
Fossil minium age

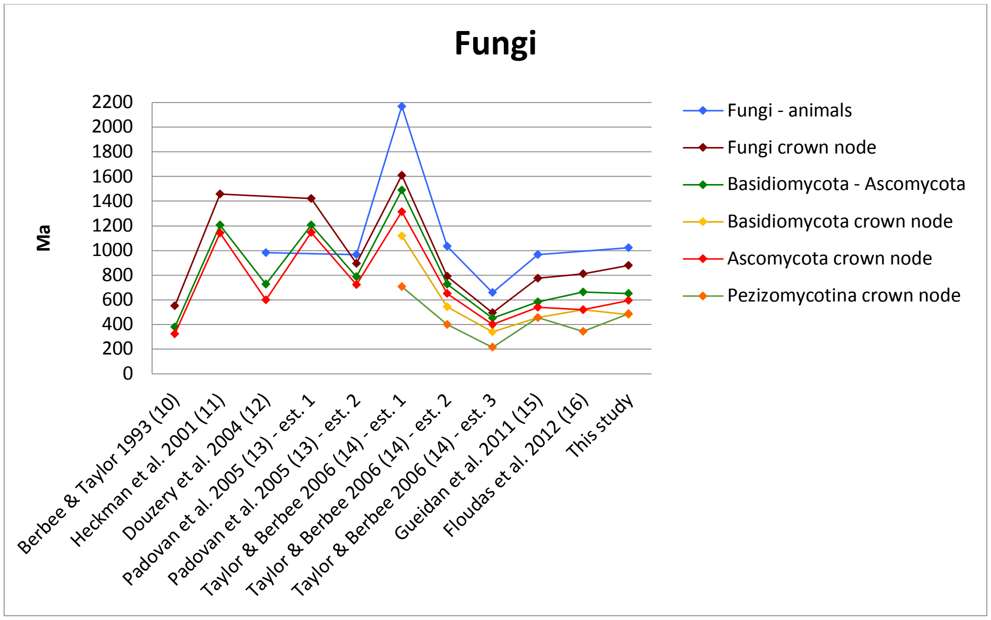
Fungi
Ma
Fungi - animals
Fungi crown node
Basidiomycota - Ascomycota
Basidiomycota crown node
Ascomycota crown node
Pezizomycotina crown node
Berbee & Taylor 1993 (10)
Heckman et al. 2001 (11)
Douzery et al. 2004 (12)
Padovan et al. 2005 (13) - est. 1
Padovan et al. 2005 (13) - est. 2
Taylor & Berbee 2006 (14) - est. 1
Taylor & Berbee 2006 (14) - est. 2
Taylor & Berbee 2006 (14) - est. 3
Gueidan et al. 2011 (15)
Floudas et al. 2012 (16)
This study

Supplementary Figure 1. Variation in estimation of divergence time for plants and fungi. The accuracy of absolute divergence times estimated with molecular clocks depends heavily on the reliability of independent temporal calibrations[17], which are most frequently obtained from the fossil record. For any biological group, the availability of useful fossil calibrations depends on the combination of biological factors responsible for the evolution of distinctive morphological features that allow unequivocal clade recognition, and of taphonomic factors that regulate the probability that these distinctive features become preserved as fossils. The utility of available fossils as calibrations also depends on the density of sampling in a phylogenetic tree. Insufficient sampling may preclude accurate recognition of the fossil's phylogenetic position. However, even if its phylogenetic relationship is correctly recognized, its associated temporal information may become irrelevant as the fossil will be placed in a long branch whose subtending node will be much older than the minimum age that the fossil provides. Estimates of most divergence times for land plants have been stable since 2005. This convergence of estimates from various studies can be explained in part by the number, distribution through time, and the taphonomic quality of the fossil record for plants. For fungi, estimations of absolute divergence time have varied greatly among studies. Overall, data sets leaned more heavily toward using multiple loci and few taxa than vice versa. The number of species included in studies published before 2007 ranged from 15 species when based on many genes, to usually fewer than 50 species when based on a single locus (nrRNA gene). The various combinations of using a relatively low number of taxa, low number of genes, and divergence time estimation methods assuming a molecular clock led to drastically different estimations across mycological studies published pre-2007. Here we estimated divergence time based on a data set of six gene regions for nearly 200 fungal species[18], with representation of fungal phyla more or less proportional to their species richness. Divergence times were estimated using a Bayesian approach[19] and taking into consideration all available fossil records (see Supplementary Note 1). Our estimated divergence times for the fungi converged on the estimations obtained independently from three other studies[14-16]. The recent convergence of divergence time estimates for fungi is a combination of the availability of more comprehensive data sets, in terms of both taxon and locus sampling, and better analytical methods[19]. Numbers in parentheses following each citation along the X axes refer to the order in which they appear in the list of Supplementary References.

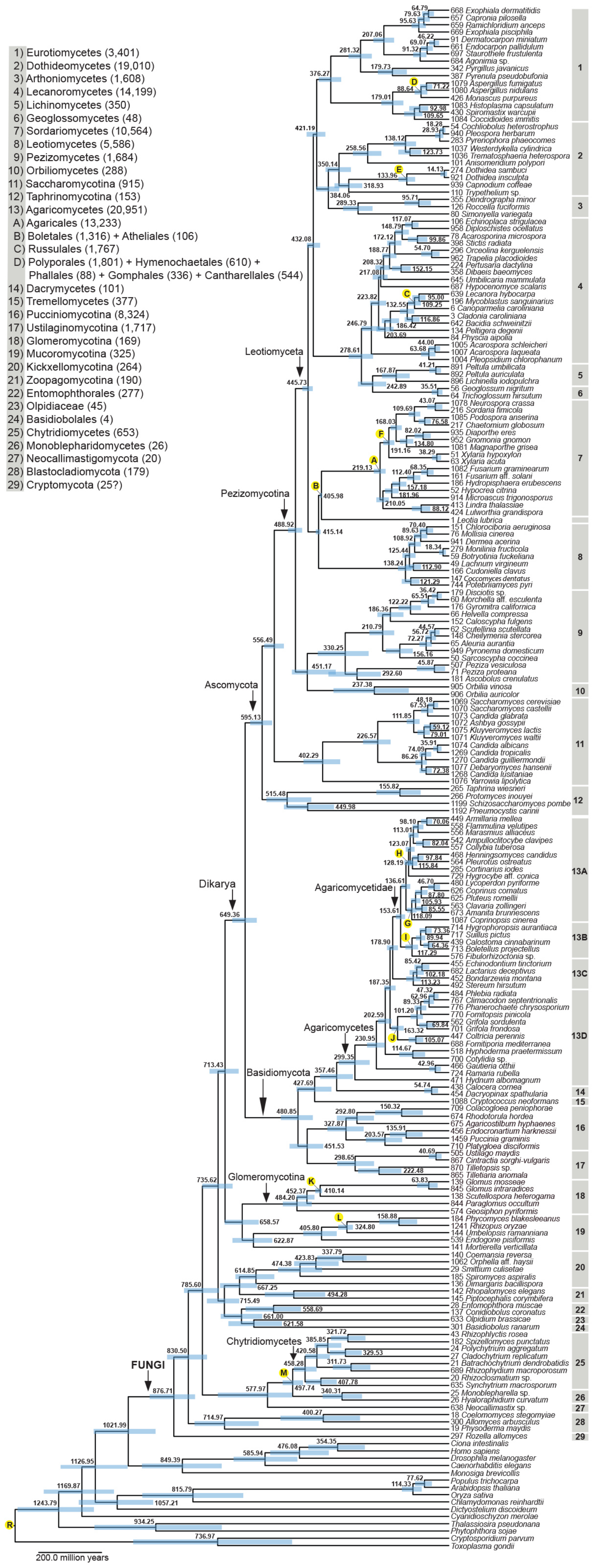

1) Eurotiomycetes (3,401)
2) Dothideomycetes (19,010)
3) Arthoniomycetes (1,608)
4) Lecanoromycetes (14,199)
5) Lichinomycetes (350)
6) Geoglossomycetes (48)
7) Sordariomycetes (10,564)
8) Leotiomycetes (5,586)
9) Pezizomycetes (1,684)
10) Orbiliomycetes (288)
11) Saccharomycotina (915)
12) Taphrinomycotina (153)
13) Agaricomycetes (20,951)
A) Agaricales (13,233)
B) Boletales (1,316) + Atheliales (106)
C) Russulales (1,767)
D) Polyporales (1,801) + Hymenochaetales (610) + Phallales (88) + Gomphales (336) + Cantharellales (544)
14) Dacrymycetes (101)
15) Tremellomycetes (377)
16) Pucciniomycotina (8,324)
17) Ustilaginomycotina (1,717)
18) Glomeromycotina (169)
19) Mucoromycotina (325)
20) Kickxellomycotina (264)
21) Zoopagomycotina (190)
22) Entomophthorales (277)
23) Olpidiaceae (45)
24) Basidiobolales (4)
25) Chytridiomycetes (653)
26) Monoblepharidomycetes (26)
27) Neocallimastigomycota (20)
28) Blastocladiomycota (179)
29) Cryptomycota (25?)
Leotiomyceta
Pezizomycotina
Ascomycota
Dikarya
Agaricomycetidae
Agaricomycetes
Basidiomycota
Glomeromycotina
Chytridiomycetes
FUNGI
668 Exophiala dermatitidis
657 Capronia pilosella
659 Ramichloridium anceps
669 Exophiala pisciphila
91 Dermatocarpon miniatum
661 Endocarpon pallidulum
697 Staurothele frustulenta
684 Agonimia sp.
342 Pyrgillus javanicus
387 Pyrenula pseudobufonia
1079 Aspergillus fumigatus
1080 Aspergillus nidulans
426 Monascus purpureus
1083 Histoplasma capsulatum
430 Spiromastix warcupii
1084 Coccidioides immitis
54 Cochliobolus heterostrophus
940 Pleospora herbarum
283 Pyrenophora phaeocomes
1037 Westerdykella cylindrica
1036 Trematosphaeria heterospora
101 Anisomeridium polypori
274 Dothidea sambuci
921 Dothidea insculpta
939 Capnodium coffeae
110 Trypethelium sp.
355 Dendrographa minor
126 Roccella fuciformis
80 Simonyella variegata
106 Echinoplaca strigulacea
958 Diploschistes ocellatus
78 Acarosporina microspora
398 Stictis radiata
296 Orceolina kerguelensis
962 Trapelia placodioides
224 Pertusaria dactylina
358 Dibaeis baeomyces
645 Umbilicaria mammulata
687 Hypocenomyce scalaris
639 Lecanora hybocarpa
196 Mycoblastus sanguinarius
6 Canoparmelia caroliniana
3 Cladonia caroliniana
642 Bacidia schweinitzii
134 Peltigera degenii
84 Physcia aipolia
1005 Acarospora schleicheri
1007 Acarospora laqueata
1004 Pleopsidium chlorophanum
891 Peltula umbilicata
892 Peltula auriculata
896 Lichinella iodopulchra
56 Geoglossum nigritum
64 Trichoglossum hirsutum
1078 Neurospora crassa
216 Sordaria fimicola
1085 Podospora anserina
217 Chaetomium globosum
935 Diaporthe eres
952 Gnomonia gnomon
1081 Magnaporthe grisea
51 Xylaria hypoxylon
63 Xylaria acuta
1082 Fusarium graminearum
161 Fusarium aff. solani
186 Hydropisphaera erubescens
52 Hypocrea citrina
914 Microascus trigonosporus
413 Lindra thalassiae
424 Lulworthia grandispora
1 Leotia lubrica
151 Chlorociboria aeruginosa
76 Mollisia cinerea
941 Dermea acerina
279 Monilinia fructicola
59 Botryotinia fuckeliana
49 Lachnum virgineum
166 Cudoniella clavus
147 Coccomyces dentatus
744 Potebniamyces pyri
179 Disciotis sp.
60 Morchella aff. esculenta
176 Gyromitra californica
66 Helvella compressa
152 Caloscypha fulgens
62 Scutellinia scutellata
148 Cheilymenia stercorea
65 Aleuria aurantia
949 Pyronema domesticum
50 Sarcoscypha coccinea
507 Peziza vesiculosa
71 Pezizia proteana
181 Ascobolus crenulatus
905 Orbilia vinosa
906 Orbilia auricolor
1069 Saccharomyces cerevisiae
1070 Saccharomyces castellii
1073 Candida glabrata
1072 Ashbya gossypii
1075 Kluyveromyces lactis
1071 Kluyveromyces waltii
1074 Candida albicans
1269 Candida tropicalis
1270 Candida guilliermondii
1077 Debaryomyces hansenii
1268 Candida lusitaniae
1076 Yarrowia lipolytica
265 Taphrina wiesneri
266 Protomyces inouyei
1199 Schizosaccharomyces pombe
1192 Pneumocystis carinii
449 Armillaria mellea
558 Flammulina velutipes
556 Marasmius alliaceus
542 Ampulloclitocybe clavipes
557 Collybia tuberosa
468 Henningsomyces candidus
564 Pleurotus ostreatus
285 Cortinarius iodes
729 Hygrocybe aff. conica
480 Lycoperdon pyriforme
626 Coprinus comatus
625 Pluteus romellii
563 Clavaria zollingeri
673 Amanita brunnescens
1087 Coprinopsis cinerea
714 Hygrophoropsis aurantiaca
717 Suillus pictus
439 Calostoma cinnabarinum
713 Boletellus projectellus
576 Fibulorhizoctonia sp.
455 Echinodontium tinctorium
682 Lactarius deceptivus
452 Bondarzewia montana
492 Stereum hirsutum
484 Phlebia radiata
767 Climacodon septentrionalis
776 Phanerochaete chrysosporium
770 Fomitopsis pinicola
562 Grifola sordulenta
701 Grifola frondosa
447 Coltricia perennis
688 Fomitiporia mediterranea
518 Hyphoderma praetermissum
700 Cotylidia sp.
466 Gautieria otthii
724 Ramaria rubella
471 Hydnum albomagnum
438 Calocera cornea
454 Dacryopinax spathularia
1088 Cryptococcus neoformans
709 Colacogloea peniophorae
674 Rhodotorula hordea
675 Agaricostilbum hyphaenes
456 Endocronartium harknessii
1459 Puccinia graminis
710 Platygloea disciformis
505 Ustilago maydis
867 Cintractia sorghi-vulgaris
870 Tilletiopsis sp.
865 Tilletiaria anomala
139 Glomus mosseae
845 Glomus intraradices
138 Scutellospora heterogama
844 Paraglomus occultum
574 Geosiphon pyriformis
184 Phycomyces blakesleeanus
1241 Rhizopus oryzae
144 Umbelopsis ramanniana
539 Endogone pisiformis
141 Mortierella verticillata
140 Coemansia reversa
1062 Orphella aff. haysii
29 Smittium culisetae
185 Spiromyces aspiralis
136 Dimargaris bacillispora
142 Rhopalomyces elegans
145 Piptocephalis corymbifera
28 Entomophthora muscae
137 Conidiobolus coronatus
633 Olpidium brassicae
301 Basidiobolus ranarum
43 Rhizophlyctis rosea
182 Spizellomyces punctatus
24 Polychytrium aggregatum
27 Cladochytrium replicatum
21 Batrachochytrium dendrobatidis
689 Rhizophydium macroporosum
20 Rhizoclosmatium sp.
635 Synchytrium macrosporum
25 Monoblepharella sp.
26 Hyaloraphidium curvatum
638 Neocallimastix sp.
18 Coelomomyces stegomyiae
300 Allomyces arbusculus
19 Physoderma maydis
297 Rozella allomyces
Ciona intestinalis
Homo sapiens
Drosophila melanogaster
Caenorhabditis elegans
Monosiga brevicollis
Populus trichocarpa
Arabidopsis thaliana
Oryza sativa
Chlamydomonas reinhardtii
Dictyostelium discoideum
Cyanidioschyzon merolae
Thalassiosira pseudonana
Phytophthora sojae
Cryptosporidium parvum
Toxoplasma gondii
200.0 million years

Supplementary Figure 2. Detailed chronogram for fungi, depicting median divergence times and associated error (95% highest posterior density) for all nodes. The names of all representative species selected for this study, preceded by their Assembling the Fungal Tree of Life (AFToL) numbers, are shown at the tips of the tree. Numbers with grey shading refer to monophyletic groups listed to the left of the tree. Each taxon name listed on the left of the tree is followed by its known species-richness (in parentheses). Nodes labeled A to M were constrained with age priors as described in the Methods and Supplementary Note 1. The node labeled R indicates the root of the tree.

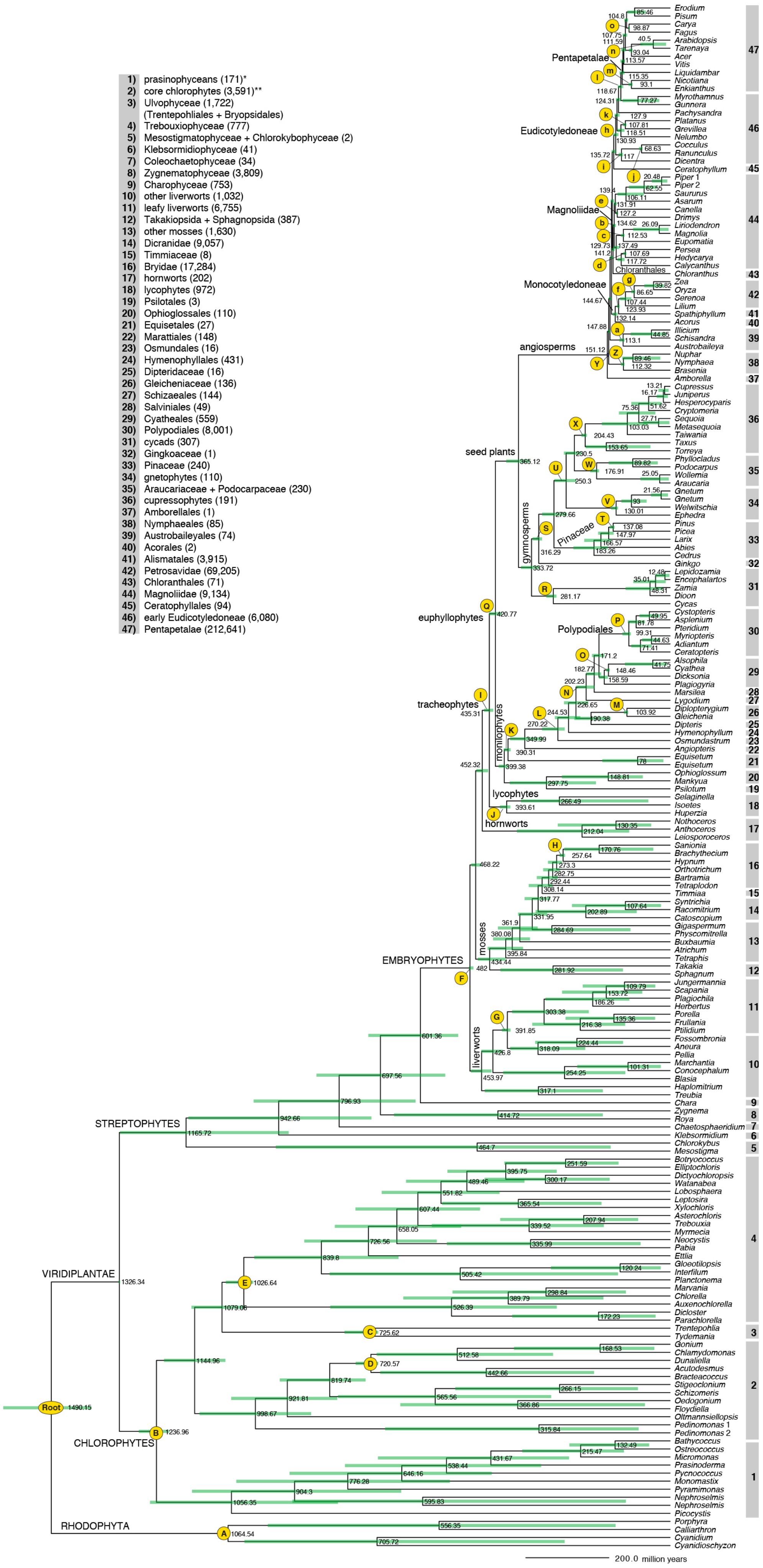

1) prasinophyceans (171)*
2) core chlorophytes (3,591)**
3) Ulvophyceae (1,722) (Trentepohliales + Bryopsidales)
4) Trebouxiophyceae (777)
5) Mesostigmatophyceae + Chlorokybophyceae (2)
6) Klebsormidiophyceae (41)
7) Coleochaetophyceae (34)
8) Zygnematophyceae (3,809)
9) Charophyceae (753)
10) other liverworts (1,032)
11) leafy liverworts (6,755)
12) Takakiopsida + Sphagnopsida (387)
13) other mosses (1,630)
14) Dicranidae (9,057)
15) Timmiaceae (8)
16) Bryidae (17,284)
17) hornworts (202)
18) lycophytes (972)
19) Psilotales (3)
20) Ophioglossales (110)
21) Equisetales (27)
22) Marattiales (148)
23) Osmundales (16)
24) Hymenophyllales (431)
25) Dipteridaceae (16)
26) Gleicheniaceae (136)
27) Schizaeales (144)
28) Salviniales (49)
29) Cyatheales (559)
30) Polypodiales (8,001)
31) cycads (307)
32) Gingkoaceae (1)
33) Pinaceae (240)
34) gnetophytes (110)
35) Araucariaceae + Podocarpaceae (230)
36) cupressophytes (191)
37) Amborellales (1)
38) Nymphaeales (85)
39) Austrobaileyales (74)
40) Acorales (2)
41) Alismatales (3,915)
42) Petrosavidae (69,205)
43) Chloranthales (71)
44) Magnoliidae (9,134)
45) Ceratophyllales (94)
46) early Eudicotyledoneae (6,080)
47) Pentapetalae (212,641)
Pentapetalae
Eudicotyledoneae
Magnoliidae
Chloranthales
Monocotyledoneae
angiosperms
seed plants
gymnosperms
Pinaceae
euphyllophytes
Polypodiales
tracheophytes
monilophytes
lycophytes
hornworts
mosses
EMBRYOPHYTES
liverworts
STREPTOPHYTES
VIRIDIPLANTAE
Root
CHLOROPHYTES
RHODOPHYTA
Erodium
Pisum
Carya
Fagus
Arabidopsis
Tarenaya
Acer
Vitis
Liquidambar
Nicotiana
Enkianthus
Myrothamnus
Gunnera
Pachysandra
Platanus
Grevillea
Nelumbo
Cocculus
Ranunculus
Dicentra
Ceratophyllum
Piper 1
Piper 2
Saururus
Asarum
Canella
Drimys
Liriodendron
Magnolia
Eupomatia
Persea
Hedycarya
Calycanthus
Chloranthus
Zea
Oryza
Serenoa
Lilium
Spathiphyllum
Acorus
Illicium
Schisandra
Austrobaileya
Nuphar
Nymphaea
Brasenia
Amborella
Cupressus
Juniperus
Hesperocyparis
Cryptomeria
Sequoia
Metasequoia
Taiwania
Taxus
Torreya
Phyllocladus
Podocarpus
Wollemia
Araucaria
Gnetum
Gnetum
Welwitschia
Ephedra
Pinus
Picea
Larix
Abies
Cedrus
Ginkgo
Lepidozamia
Encephalartos
Zamia
Dioon
Cycas
Cystopteris
Asplenium
Pteridium
Myriopteris
Adiantum
Ceratopteris
Alsophila
Cyathea
Dicksonia
Plagiogyria
Marsilea
Lygodium
Diplopterygium
Gleichenia
Dipteris
Hymenophyllum
Osmundastrum
Angiopteris
Equisetum
Equisetum
Ophioglossum
Mankyua
Psilotum
Selaginella
Isoetes
Huperzia
Nothoceros
Anthoceros
Leiosporoceros
Sanionia
Brachythecium
Hypnum
Orthotrichum
Bartramia
Tetraplodon
Timmiaa
Syntrichia
Racomitrium
Catoscopium
Gigaspermum
Physcomitrella
Buxbaumia
Atrichum
Tetraphis
Takakia
Sphagnum
Jungermannia
Scapania
Plagiochila
Herbertus
Porella
Frullania
Ptilidium
Fossombronia
Aneura
Pellia
Marchantia
Conocephalum
Blasia
Haplomitrium
Treubia
Chara
Zygnema
Roya
Chaetosphaeridium
Klebsormidium
Chlorokybus
Mesostigma
Botryococcus
Elliptochloris
Dictyochloropsis
Watanabea
Lobosphaera
Leptosira
Xylochloris
Asterochloris
Trebouxia
Myrmecia
Neocystis
Pabia
Ettlia
Gloeotilopsis
Interfilum
Planctonema
Marvania
Chlorella
Auxenochlorella
Dicloster
Parachlorella
Trentepohlia
Tydemania
Gonium
Chlamydomonas
Dunaliella
Acutodesmus
Bracteacoccus
Stigeoclonium
Schizomeris
Oedogonium
Floydiella
Oltmannsiellopsis
Pedinomonas 1
Pedinomonas 2
Bathycoccus
Ostreococcus
Micromonas
Prasinoderma
Pycnococcus
Monomastix
Pyramimonas
Nephroselmis
Nephroselmis
Picocystis
Porphyra
Calliarthron
Cyanidium
Cyanidioschyzon
200.0 million years

Supplementary Figure 3. Detailed chronogram for plants, depicting median divergence times and associated error (95% highest posterior density). Numbers with grey shading refer to monophyletic groups listed to the left of the tree, followed by their species richness (in parentheses). Nodes labeled A to Z and a to o were constrained with age priors as described in the Methods and Supplementary Note 2.

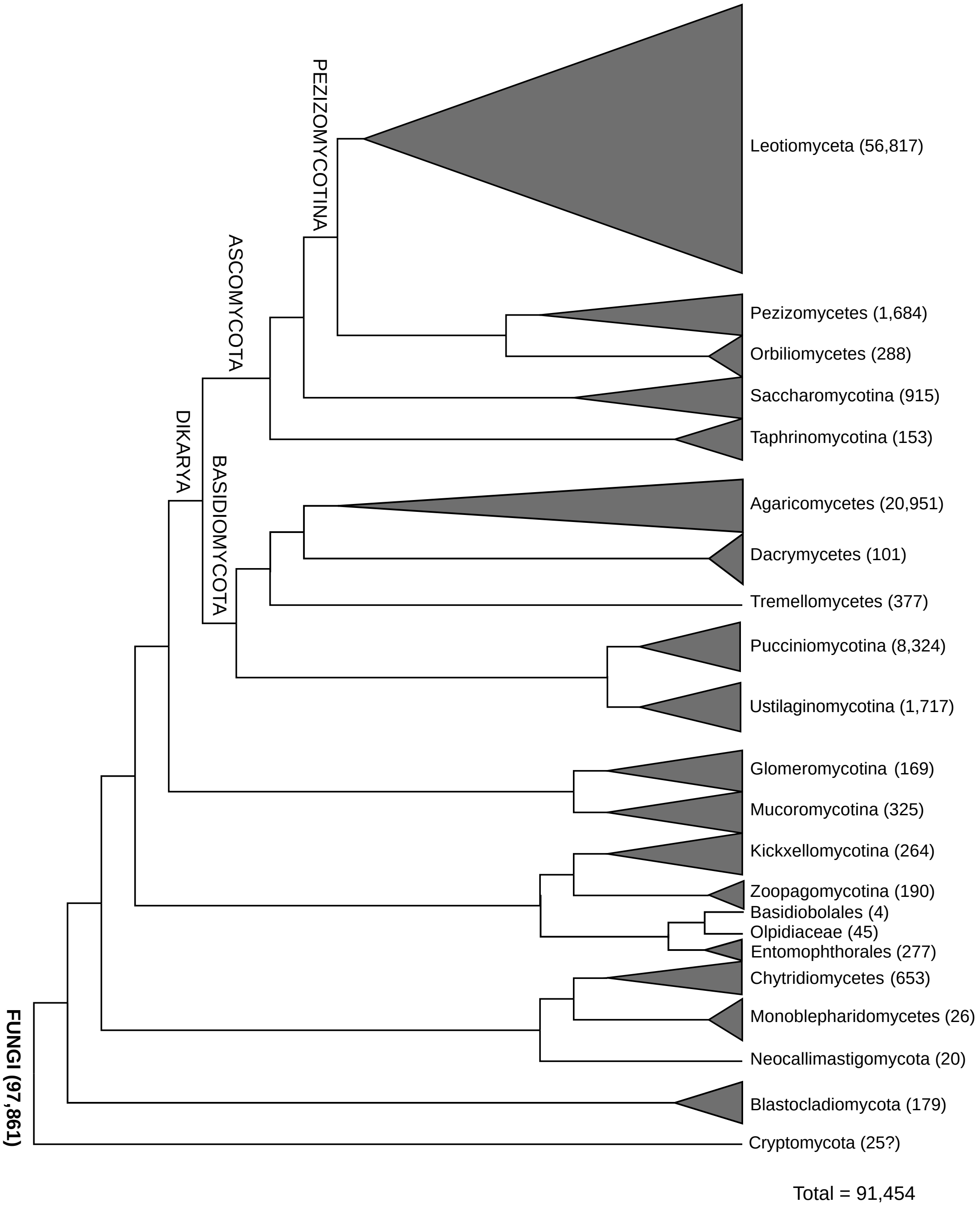

Supplementary Figure 4. Topology of fungi, indicating the number of species assigned to each monophyletic group[20], and used in the diversification analysis in MEDUSA[21]. The height of the terminal cones is proportional to the number of species included in Fig. 1.

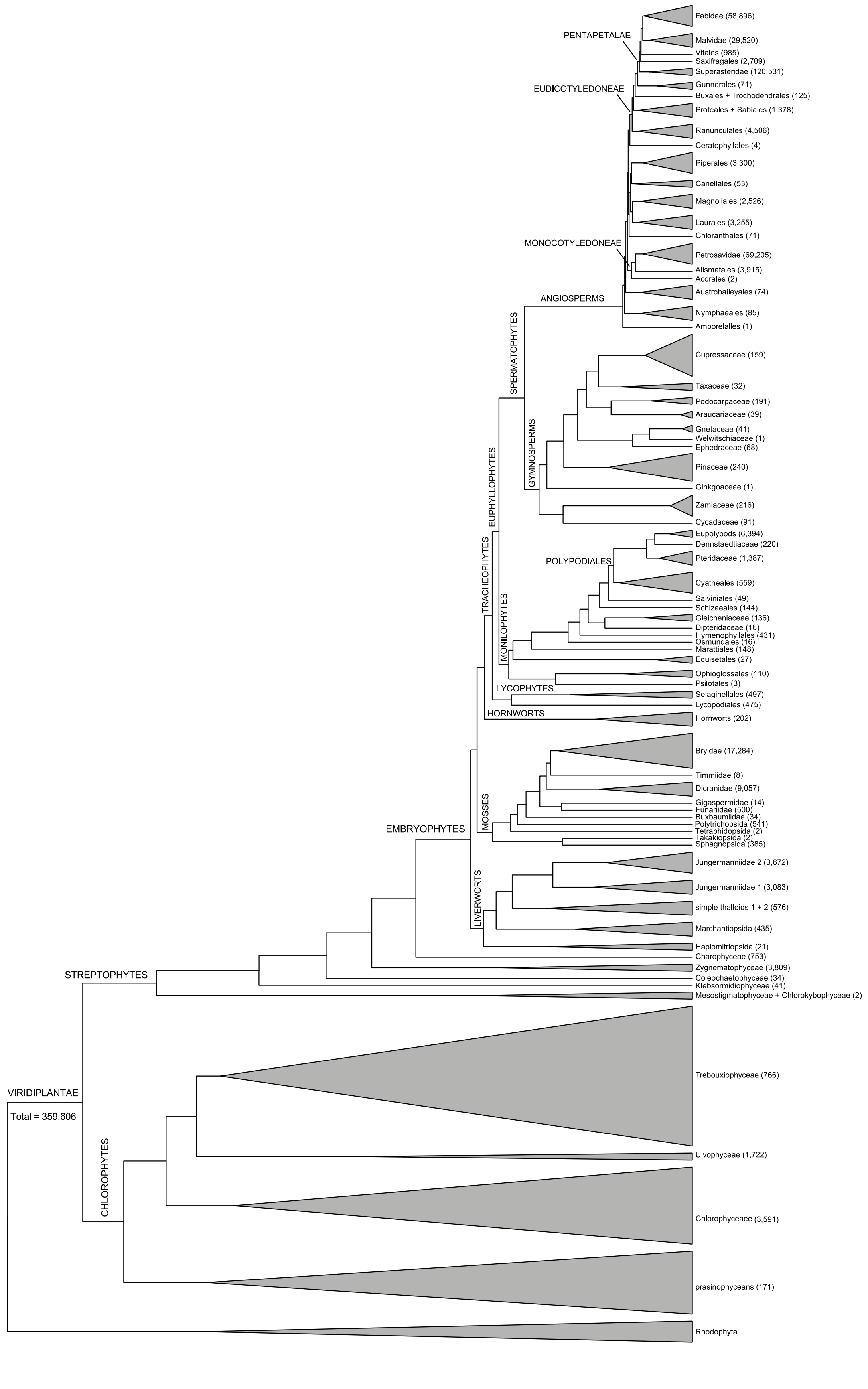

Fabidae (58,896)
PENTAPETALAE
Malvidae (29,520)
Vitales (985)
Saxifragales (2,709)
Superasteridae (120,531)
Gunnerales (71)
EUDICOTYLEDONEAE
Buxales + Trochodendrales (125)
Proteales + Sabiales (1,378)
Ranunculales (4,506)
Ceratophyllales (4)
Piperales (3,300)
Canellales (53)
Magnoliales (2,526)
Laurales (3,255)
Chloranthales (71)
MONOCOTYLEDONEAE
Petrosavidae (69,205)
Alismatales (3,915)
Acorales (2)
ANGIOSPERMS
Austrobaileyales (74)
Nymphaeales (85)
Amborelalles (1)
SPERMATOPHYTES
Cupressaceae (159)
Taxaceae (32)
Podocarpaceae (191)
Araucariaceae (39)
Gnetaceae (41)
Welwitschiaceae (1)
Ephedraceae (68)
GYMNOSPERMS
Pinaceae (240)
EUPHYLLOPHYTES
Ginkgoaceae (1)
Zamiaceae (216)
Cycadaceae (91)
TRACHEOPHYTES
Eupolypods (6,394)
Dennstaedtiaceae (220)
POLYPODIALES
Pteridaceae (1,387)
Cyatheales (559)
MONILOPHYTES
Salviniales (49)
Schizaeales (144)
Gleicheniaceae (136)
Dipteridaceae (16)
Hymenophyllales (431)
Osmundales (16)
Marattiales (148)
Equisetales (27)
Ophioglossales (110)
Psilotales (3)
LYCOPHYTES
Selaginellales (497)
Lycopodiales (475)
HORNWORTS
Hornworts (202)
Bryidae (17,284)
Timmiidae (8)
Dicranidae (9,057)
MOSSES
Gigaspermidae (14)
Funariidae (500)
Buxbaumiidae (34)
Polytrichopsida (541)
Tetraphidopsida (2)
Takakiopsida (2)
Sphagnopsida (385)
EMBRYOPHYTES
Jungermanniidae 2 (3,672)
Jungermanniidae 1 (3,083)
LIVERWORTS
simple thalloids 1 + 2 (576)
Marchantiopsida (435)
Haplomitriopsida (21)
Charophyceae (753)
Zygnematophyceae (3,809)
STREPTOPHYTES
Coleochaetophyceae (34)
Klebsormidiophyceae (41)
Mesostigmatophyceae + Chlorokybophyceae (2)
Trebouxiophyceae (766)
VIRIDIPLANTAE
Total = 359,606
CHLOROPHYTES
Ulvophyceae (1,722)
Chlorophyceaee (3,591)
prasinophyceans (171)
Rhodophyta

Supplementary Figure 5. Topology of plants, indicating the number of species assigned to each monophyletic group[22-26], and used in the diversification analysis with MEDUSA[21]. The height of the terminal cones is proportional to the number of species included in Fig. 1.

Supplementary Table 1. Estimated species richness[20], median age, and 95% highest posterior density (HPD) of major fungal clades (crown nodes). Numbers in the first column correspond to clades listed in Fig. 1. These results can also be visualized in Supplementary Fig. 2. The number of species in the recently published phylum Cryptomycota is not known, hence the number represents a best estimate.

Number	Clade	Number of species	Median age (Ma)	Age 95% HPD (Ma)
1	Eurotiomycetes	3,401	376.27	342.92-414.92
2	Dothideomycetes	19,010	350.14	311.98-393.00
3	Arthoniomycetes	1,608	289.33	242.77-337.50
4	Lecanoromycetes	14,199	246.79	218.51-279.59
5	Lichinomycetes	350	167.87	124.73-213.27
6	Geoglossomycetes	48	35.51	20.08-54.56
7	Sordariomycetes	10,564	219.14	201.33-239.61
8	Leotiomycetes	5,586	138.24	118.34-162.18
9	Pezizomycetes	1,684	330.25	255.15-417.91
10	Orbiliomycetes	288	237.38	127.28-367.86
11	Saccharomycotina	915	402.29	346.03-463.77
12	Taphrinomycotina	153	515.48	437.97-581.72
13*	Agaricomycetes	20,951	299.35	251.93-350.94
13A	Agaricales	13,233	136.61	122.66-151.73
13B	Boletales+Atheliales	1,316+106	117.29	97.72-138.54
13C	Russulales	1,767	113.23	89.35-136.26
14	Dacrymycetes	101	54.74	34.03-78.68
15	Tremellomycetes	377	427.69	374.05-486.09
16	Pucciniomycotina	8,324	327.87	271.22-387.64
17	Ustilaginomycotina	1,717	298.65	237.95-356.20
18	Glomeromycotina	169	484.20	436.96-529.49
19	Mucoromycotina	325	622.87	562.56-689.68
21	Kickxellomycotina	264	614.85	555.99-680.30
21	Zoopagomycotina	190	494.28	393.69-586.68
22	Entomophthorales	277	558.69	471.29-648.33
23+24	Olpidiaceae+Basidiobolales	45+4	621.58	531.81-703.06
25	Chytridiomycetes	653	458.29	421.49-494.09
26	Monoblepharidomycetes	26	340.31	276.41-402.26
27	Neocallimastigomycota	20	577.97	499.45-736.40
28	Blastocladiomycota	179	714.97	605.83-810.99
29	Cryptomycota	25 ?	876.71	808.71-954.02

* 13D (in Fig. 1 and Supplementary Fig. 2) is a paraphyletic group including Polyporales (1,801) + Hymenochaetales (610) + Phallales (88) + Gomphales (336) + Cantharellales (544). It was not included in this table because its divergence time is the same as for Agaricomycetes, node 13.

Supplementary Table 2. Estimated species richness[22-26], median age, and 95% highest posterior density (HPD) of major plant clades (crown nodes). These results can also be visualized in Supplementary Fig. 3.

Clade	Number of species	Median age (Ma)	Age 95% HPD (Ma)
Embryophytes (land plants)	300,781	482.00	485.00 – 473.28
Tracheophytes (vascular plants)	283,656	435.31	448.53 – 425.73
Euphyllophytes	282,456	420.77	436.03 – 404.69
Monilophytes (ferns)	9,118	399.38	413.17 – 388.80
Spermatophytes (seed plants)	273,338	365.12	394.90 – 338.00
Cycadophyta (cycads)	210	281.17	298.35 – 273.44
Gnetophyta (gnetophytes)	71	130.01	142.54 – 125.50
Pinaceae	225	183.26	233.35 – 149.97
Cupressophyta (cupressophytes)	363	230.50	256.01 – 213.00
Angiosperms (flowering plants)	272,468	151.12	151.80 – 148.79
Mesangiospermae	272,293	141.90	144.87 – 136.88
Magnoliidae	9,999	134.62	138.99 – 130.43
Monocotyledoneae (monocots)	62,118	132.14	138.41 – 126.01
Eudicotyledoneae (eudicots)	200,095	130.93	136.76 – 124.34
Pentapetalae	193,510	115.35	121.93 – 109.66

Supplementary Table 3. Absolute position of mutational hotspots (ambiguous alignments) in each character set. Size of each characters set is given in the second line as #chars (nt).

Hotspots	cpLSU	cpSSU	*atp*B	*mat*K	*psa*A	*psb*B	*rbc*L	*rps*4	*trn*L
#chars	1140	623	25	0	0	1	0	111	2388
H1	324-347	100-165	10-30	N.A.	N.A.	958	N.A.	283-393	42-549
H2	438-759	335-381	1642-1645	N.A.	N.A.	N.A.	N.A.	N.A.	631-1032
H3	907-1084	954-1041	N.A.	N.A.	N.A.	N.A.	N.A.	N.A.	1057-1065
H4	1849-1874	1155-1223	N.A.	N.A.	N.A.	N.A.	N.A.	N.A.	1155-1489
H5	2069-2153	1554-1594	N.A.	N.A.	N.A.	N.A.	N.A.	N.A.	1524-2302
H6	2552-2811	1749-2028	N.A.	N.A.	N.A.	N.A.	N.A.	N.A.	2331-2682
H7	3361-3447	2350-2381	N.A.	N.A.	N.A.	N.A.	N.A.	N.A.	2804-2808
H8	3807-3838	N.A.	N.A.	N.A.	N.A.	N.A.	N.A.	N.A.	N.A.
H9	4011-4053	N.A.	N.A.	N.A.	N.A.	N.A.	N.A.	N.A.	N.A.
H10	4203-4252	N.A.	N.A.	N.A.	N.A.	N.A.	N.A.	N.A.	N.A.
H11	5294-5326	N.A.	N.A.	N.A.	N.A.	N.A.	N.A.	N.A.	N.A.

Supplementary Table 4. Sequence and alignment statistics of each marker (charset), including the number of characters provided by each marker (#chars) with and without mutational hotspots (HS; corresponding to ambiguous alignments), the length range of the sequences for each marker, the mean and the standard deviation (S.D.), as well as the proportional contribution of the different regions to the data matrix (variable and informative site as well as GC content). The length range together with the mean and S.D. are provided from the original alignment. Variable and parsimony informative sites are presented based on the data set with the hotspots excluded.

charset	#chars excl. HS	#chars incl. HS	length range [nt]	mean [nt]	S.D.	variable [%]	informative [%]	GC [%]
LSU	4334	5474	815-3601	2725.8	290.8	52.1	37.5	52.6
SSU	1854	2477	603-1811	1458.0	116.7	56.4	34.6	54.7
*atp*B	1647	1672	958-1476	1426.4	83.3	65.8	56.5	40.6
*mat*K	2877	2877	0-1596	1457.6	215.6	62.0	54.6	32.2
*psa*A	2305	2305	429-2277	2195.4	233.8	67.7	56.0	41.4
*psb*B	1551	1552	702-1539	1478.0	111.3	71.3	60.7	42.6
*rbc*L	1473	1473	503-1467	1394.3	107.2	72.0	63.8	42.9
*rps*4	1218	1329	402-774	604.5	40.2	63.2	48.5	35.3
*trn*L	450	2838	81-1214	451.2	189.3	51.3	33.5	36.9

Supplementary Table 5. Summary of posterior distribution of differences in divergence times between selected plant and fungal clades (all ages are in millions of years; see Fig. 2).*

--Fig. 2--	---- Node ----		----------- F-P -----------		------------------- Posterior probabilities -------------------					
panel	fungal	plant	mean	95% HPD	x	|F-P| < x	F-P < x	F-P > x	P-F < x	P-F > x
Fig. 2a	F1	P1	-11.0858	(-178.761,159.063)						
					1	0.008769	0.552907	0.447093	0.455862	0.544138
					5	0.043816	0.570430	0.429570	0.473386	0.526614
					10	0.087472	0.592222	0.407778	0.495250	0.504750
					20	0.173918	0.634957	0.365043	0.538961	0.461039
					40	0.340292	0.714822	0.285178	0.625470	0.374530
					60	0.489610	0.784978	0.215022	0.704633	0.295367
Fig. 2b	F1	P2	-17.9722	(-90.5377,50.8387)						
					1	0.022431	0.698579	0.301421	0.323852	0.676148
					5	0.111841	0.741405	0.258595	0.370437	0.629563
					10	0.221736	0.790188	0.209812	0.431548	0.568452
					20	0.426443	0.869972	0.130028	0.556471	0.443529
					40	0.734159	0.962883	0.037117	0.771276	0.228724
					60	0.885495	0.991942	0.008058	0.893553	0.106447
Fig. 2c	F2	P3	3.5442	(-45.1035,49.3131)						
					1	0.033655	0.460252	0.539748	0.573403	0.426597
					5	0.166171	0.528228	0.471772	0.637943	0.362057
					10	0.321779	0.611469	0.388531	0.710310	0.289690

					20	0.585590	0.757167	0.242833	0.828423	0.171577
					40	0.895015	0.930523	0.069477	0.964492	0.035508
					60	0.985993	0.986650	0.013350	0.999343	0.000657
Fig. 2d	F3	P3	8.9087	(-14.0979,34.358)						
					1	0.055002	0.276055	0.723945	0.778946	0.221054
					5	0.270675	0.399885	0.600115	0.870790	0.129210
					10	0.508231	0.561276	0.438724	0.946955	0.053045
					20	0.811321	0.814937	0.185063	0.996384	0.003616
					40	0.987544	0.987544	0.012456	1.000000	0.000000
					60	0.999745	0.999745	0.000255	1.000000	0.000000
Fig. 2e	F4	P4	13.3507	(-7.79089,35.5112)						
					1	0.035975	0.127124	0.872876	0.908850	0.091150
					5	0.180909	0.223667	0.776333	0.957241	0.042759
					10	0.373854	0.387334	0.612666	0.986520	0.013480
					20	0.736032	0.736671	0.263329	0.999361	0.000639
					40	0.988380	0.988380	0.011620	1.000000	0.000000
					60	0.999987	0.999987	0.000013	1.000000	0.000000
Fig. 2f	F5	P3	-43.9008	(-71.4114,-11.2798)						
					1	0.001233	0.998125	0.001875	0.003109	0.996891
					5	0.007300	0.999274	0.000726	0.008027	0.991973
					10	0.020314	0.999850	0.000150	0.020465	0.979535
					20	0.077374	1.000000	0.000000	0.077375	0.922625
					40	0.365973	1.000000	0.000000	0.365973	0.634027

					60	0.863221	1.000000	0.000000	0.863221	0.136779
Fig. 2g	F6	P5	-65.1869	(-123.707,-6.79377)						
					1	0.002730	0.981869	0.018131	0.020860	0.979140
					5	0.014008	0.986338	0.013662	0.027670	0.972330
					10	0.029617	0.990582	0.009418	0.039035	0.960965
					20	0.067699	0.995758	0.004242	0.071940	0.928060
					40	0.198029	0.999450	0.000550	0.198579	0.801421
					60	0.419810	0.999976	0.000024	0.419834	0.580166
Fig. 2h	F6	P6	-35.0941	(-91.0421,20.86)						
					1	0.012361	0.891993	0.108007	0.120369	0.879631
					5	0.062113	0.913847	0.086153	0.148267	0.851733
					10	0.125383	0.936227	0.063773	0.189156	0.810844
					20	0.259291	0.967234	0.032766	0.292057	0.707943
					40	0.549495	0.992967	0.007033	0.556528	0.443472
					60	0.803328	0.998921	0.001079	0.804407	0.195593
Fig. 2i	F6	P7	113.5777	(45.6821,181.494)						
					1	0.000208	0.000960	0.999040	0.999249	0.000751
					5	0.001088	0.001541	0.998459	0.999547	0.000453
					10	0.002318	0.002535	0.997465	0.999784	0.000216
					20	0.005449	0.005482	0.994518	0.999967	0.000033
					40	0.020789	0.020795	0.979205	0.999995	0.000005
					60	0.063404	0.063404	0.936596	1.000000	0.000000
Fig. 2j	F7	P7	-6.6426	(-58.8765,37.5673)						

					1	0.034678	0.581088	0.418912	0.453590	0.546410
					5	0.171411	0.649901	0.350099	0.521511	0.478489
					10	0.333705	0.732783	0.267217	0.600922	0.399078
					20	0.608090	0.872655	0.127345	0.735435	0.264565
					40	0.889896	0.989965	0.010035	0.899931	0.100069
					60	0.965736	0.999837	0.000163	0.965899	0.034101
Fig. 2k	F8	P7	-33.1762	(-83.3478,9.56592)						
					1	0.014629	0.951058	0.048942	0.063570	0.936430
					5	0.073527	0.973040	0.026960	0.100487	0.899513
					10	0.150175	0.988783	0.011217	0.161392	0.838608
					20	0.320078	0.998777	0.001223	0.321302	0.678698
					40	0.659091	1.000000	0.000000	0.659091	0.340909
					60	0.864603	1.000000	0.000000	0.864603	0.135397
Fig. 2l	F8	P8	2.9233	(-12.601,18.1491)						
					1	0.089661	0.416771	0.583229	0.672890	0.327110
					5	0.434914	0.604709	0.395291	0.830206	0.169794
					10	0.759032	0.807653	0.192347	0.951379	0.048621
					20	0.979846	0.980571	0.019429	0.999275	0.000725
					40	1.000000	1.000000	0.000000	1.000000	0.000000
					60	1.000000	1.000000	0.000000	1.000000	0.000000

* The mean difference in the absolute value of the estimated fungal (F) and plant (P) ages, along with credible intervals, is shown under F-P. For each pair of clades, the right portion of the table shows the posterior probabilities that the times of fungal and plant divergences differ in absolute value, by less than, or by more than, six different amounts (x = 1, 5, 10, 20, 40, and 60 million years).

Supplementary Note 1: Fossil constraints for fungi

List of constraints on fungal tree, corresponding to yellow nodes on Supplementary Fig. 2.

Node A: Fungal bodies of the genus *Colletotrichum* were found in dung of the dinosaur *Isisaurus* from the Upper Cretaceous. The authors[27] placed the genus in the family Melanconiaceae (Deuteromycetes). Members of *Colletotrichum* are anamorphs of the genus *Glomerella*, a genus currently placed in the order Glomerellales (Sordariomycetes)[20]. Therefore, a minimum age constraint of 65.2 Ma was applied to the most recent common ancestor (MRCA) of the Sordariomycetes (see Supplementary Fig. 2). Extant *Colletotrichum* species are parasites of diverse angiosperms and some Pinaceae, with many species occurring as endophytes.

Node B: *Paleopyrenomycites devonicus* Taylor, Hass, Kerp, Krings et Hanlin was found in the cortex of aerial stems and rhizomes of the 400 Ma fossilized vascular plant *Asteroxylon* from the Rhynie Chert[28]. Due to the presence of a perithecial ascoma, an apparently unitunicate ascus, and some centrum features more in common with *Xylaria*-type centrum development, the fossil appears to have close affinities with extant pyrenomycetes (currently Sordariomycetes). A minimum age constraint of 404.2 Ma was applied to the node from which the Sordariomycetes diverged.

Node C: *Anzia electra* Rikkinen & Poinar is a lichenized fungus that was found in Baltic amber[29] dating from the Early Oligocene (55 Ma) to Late Eocene (35 Ma). Because of the exceptional preservation of organisms in amber, the fossil could be identified to the species level. The genus is placed in the family Anziaceae or Parmeliaceae (s. auct.), order Lecanorales. A minimum age constraint of 33.8 Ma was applied to the basal node of a clade including Parmeliaceae and other members of the order Lecanorales.

Node D: *Aspergillus collembolorum* Dörfelt & A.R. Schmidt was found overgrowing a springtail (suborder Entomobryomorpha) in Baltic amber[30]. Excellent preservation of the hyphae and conidiophores allowed the placement of the fossil within the genus *Aspergillus* (Trichocomaceae, Eurotiales, Eurotiomycetidae, Eurotiomycetes). A minimum age constraint of 33.8 Ma was applied to the node leading to the divergence of *Aspergillus* and *Monascus*.

Node E: *Metacapnodium succinum* (Dörfelt, A.R. Schmidt & J. Wunderl.) Rikkinen, Dörfelt, A.R. Schmidt & J. Wunderl. specimens were found in Baltic and Bitterfield amber[31]. As the Bitterfield amber was suggested to have originated by transport and re-deposition of Baltic amber during the Miocene[32-33], all fossil specimens found in Bitterfield amber should also be 55-35 Ma. Several preserved features showed similarities with extant *Metacapnodium* (Metacapnodiaceae, Capnodiales, Dothideomycetes) and supported the placement of the fossils within that genus. A minimum age constraint of 33.8 Ma was applied to the basal node of a clade including Capnodiales and Dothideales.

Node F: A *Xylaria* species was found in Dominican amber dating from the Eocene to the Miocene period[34]. A minimum age constraint of 15.97 Ma was applied to the node that leads to the divergence of the Xylariales.

Node G: The gilled mushroom *Coprinites dominicana*, found in Dominican amber (15 Ma to 40 Ma), has affinities with the present-day genus *Coprinus* and was placed in the family Coprinaceae, Basidiomycota[35]. Hibbett et al. (1997)[36] interpreted *Coprinites* as either an Agaricaceae sensu Singer or a Coprinaceae. A minimum age constraint of 15.97 Ma was applied to the node that leads to the divergence of the Coprinaceae and Agaricaceae.

Node H: *Archaeomarasmius leggeti* Hibbett, Grimaldi & Donoghue from mid-Cretaceous amber of New Jersey resembles the extant genera *Marasmius* and *Marasmiellus* and was placed in the family Tricholomataceae, Agaricales, Basidiomycota[36]. A minimum age of 88.3 Ma should have been applied to the node from which the Tricholomataceae diverged, however, because that clade was not well-supported, the constraint was place on the immediate well-supported node deeper in the phylogeny.

Node I: A permineralized ectomycorrhizal fungus was found in association with roots of *Pinus* in the Princeton Chert of the Middle Eocene[37]. Several features such as presence of a Hartig net and corraloid clusters, and an absence of clamp connections on the emanating hyphae, suggested a close relation of the fungal fossil to Basidiomycota extant genera *Rhizopogon* and *Suillus*, both in the Boletales. A minimum age of 40.2 Ma was applied to the node from which the Boletales diverged.

Node J: *Quatsinoporites cranhamii* Smith, Currah et Stockey is an Early Cretaceous (Barremian) basidiomycete preserved in marine calcareous nodules[38]. It was placed in the family Hymenochaetaceae (Hymenochaetales) due to the poroid hymenophore, the presence of setae, and a monomitic hyphal system lacking clamp connections. A minimum age of 124 Ma should have been applied to the node from which the Hymenochaetaceae diverged, but because that clade was not well supported, the constraint was place on the next, deeper, well-supported clade.

Node K: *Glomites rhyniensis* Taylor, Remy, Hass et Kerp represents an arbuscular endomycorrhiza in association with the Early Devonian plant *Aglaophyton*[39]. The authors are almost certain that *Glomites* belong to the family Glomaceae (currently Glomeraceae) with several characters in common with *Glomus*. A minimum age of 404.2 Ma was applied to the node from which Glomeraceae diverged.

Node L: *Protoascon missouriensis* Batra, Segal et Baxter was found associated with the charophyte *Palaeonitella* in Middle Pennsylvanian permineralization. The fossil represents a Zygosporangium-suspensor complex of a zygomycete comparable to some modern Mucorales[40]. A minimum age of 305.5 Ma was applied to the node from which the Mucorales diverged.

Node M: *Lyonomyces pyriformis* Taylor, Hass et Remy is an aquatic fungal fossil preserved in the 400 Ma Lower Devonian Rhynie Chert[41]. It has the same morphological features as those of numerous aquatic fungi, among them *Rhizophydium* (Chytridiales). Because the systematic position of *Lyonomyces pyriformis* cannot be determined with more precision, a minimum age of 404.2 Ma was applied to the most recent common ancestor (MRCA) of the Chytridiomycetes and Monoblepharidomycetes.

Root (node R): We applied a lognormal prior distribution on the age of the root of the phylogeny that placed 90% of the prior density between 501 Ma and 1965 Ma (Supplementary Fig. 2).

Supplementary Note 2: Fossil constraints for Viridiplantae

Root: Age of the MRCA between Rhodophyta and Viridiplantae was estimated in a study of the timing of early eukaryotic diversification[42]. The tree model root height was assigned a uniform prior between 1394 and 1623 Ma, corresponding to the 95% HPD estimated for the MRCA between Rhodophyta and Viridiplantae in Tree E of Parfrey et al. (2011)[42]. This tree was estimated including the largest taxonomic sample in their analysis (109 taxa), using Proterozoic and Phanerozoic fossils as calibrations, considering the calibration for Rhodophyta (*Bangiomorpha*) to be 1174 Ma, and simultaneously estimating the root of the tree. The prior for the height of the root of the tree was obtained from a uniform distribution between 1394 and 1623 Ma.

Node A: Rhodophyta crown node calibrated with *Bangiomorpha pubescens* Butterfield, from the late Mesoproterozoic[43-44]. *Bangiomorpha pubescens* has been considered as the oldest taxonomically-resolved fossil eukaryote. Its diagnostic characters are the "fourfold radially symmetrical arrangement of wedge-shaped cells that constitute most multisertiate filaments. This arrangement documents the unique pattern of longitudinal intercalary cell division that is otherwise known only in modern *Bangia*. Likewise, the hierarchical pairing of cells in uniseriate filaments documents the bangiaceaen habit of diffuse growth whereby all vegetative cells contribute to initial filament elongation through transverse intercalary cell division (vs. the apical growth of most other algae and filamentous cyanobacteria)."[43]. *Bangiomorpha pubescens* was compared with potentially related taxa, including Cyanobacteria, Schizomeridaceae, Porphyridales and the Prasiolalean chlorophyte *Rosevingiella*, but was found to be almost indistinguishable from the modern bangiophyte red algae *Bangia*[43]. However, as noted by Yang et al. (2015)[45] "several *Bangiomorpha*-like, simple filamentous species occur among the deeply diverging Compsopogonophyceae (i.e., *Compsopogon*, *Compopogoniopsis*, *Erythrotrichia*, *Rhodochaete*) and Stylonematophyceae (i.e., *Bangiopsis*, *Purpureofilum*, *Stylonema*)". Their interpretation is that *Bangiomorpha* "associates with any one of a number of the deep red algal lineages, possibly even an extinct lineage that evolved characters in parallel to the Bangiophyceae and Compsopogonophyceae. Therefore, it would not be unreasonable to place the *Bangiomorpha* constraint as a stem taxon to the early branching lineages of red algae."[45]. *Bangiomorpha pubescens* was found in the Hunting Formation from NW Somerset Island, arctic Canada, in a Proterozoic sedimentary basin. The Hunting Formation is bracketed between 1267-723 Ma based on detailed stable isotope chemostratigraphy, and litho- and biostratigraphy correlations with Mesoproterozoic units in the nearby Baffin Island and Greenland, but a Pb-Pb dating of carbonates from the Hunting-correlative Society Cliffs formation on Baffin Island yielded a more constrained age of 1198 ± 24 Ma[42-44, 46] Note that the affinity and age of *Bangiomorpha pubescens* has been debated (e.g., Parfrey et al. 2011[42]). Its position within the stratigraphic sequence, and other factors would suggest an age closer to 1200 Ma (discussed in Knoll et al. 2013[46]), whereas a conservatively implemented calibration would require the absolute upper bound of the Hunting Formation, at 720 Ma, as a minimum age[42]. *Bangiomorpha pubescens* was used to calibrate the crown node of Rhodophyta with a lognormal prior distribution with mean = 4.3239, standard deviation = 0.75, and offset = 1000.

Node B: Prasinophytina stem node was calibrated with *Pterospermella* spp., from the late Mesoproterozoic/earliest Neoproterozoic[47]. *Pterospermella* spp. are "oval to circular vesicle consist[ing]

of a body surrounded at the equatorial plane by a membrane. The membrane is wide, slightly and transparent and the ratio of overall diameter vs. inner body diameter ranges from 1.3 to 1.8. In some specimens, irregularly distributed thick aristae, up to 99 µm in length, occur."[47]. *Pterospermella* is similar to extant Pyramimonadales species with a single large ring around the equator of the phycoma. Five specimens of *Pterospermella* were recorded from the Orlik Fjord and Kap Powell formations of the Dundas Group, and from the Qaanaaq Formation of the Baffin Bay Group, of the Thule Basin in the Canadian-Icelandic shield, which, based on biostratigraphic correlations and independent stable isotope data is of c. 1300 to c. 1200 Ma[47]. *Pterospermella* spp. were used to calibrate the stem node of Prasinophytina with a lognormal prior distribution with mean = 3.8131, standard deviation = 0.75, and offset = 1200.

Node C: Cladophorales stem node calibrated with species of *Proterocladus* Butterfield, from the late Riphean (Neoproterozoic)[48]. *Proterocladus* are "Multicellular, uniseriate and occasionally branched filaments with intercellular septa. Cells thin walled, psilate and cylindrical; length highly variable but typically much longer than wide. Branches usually subjacent to a septum in the primary axis and themselves often septate. Apical terminations simply rounded or capitate."[48]. *Proterocladus* is considered to be morphologically similar to extant Chlorophyta, in particular the branching septate filaments of *Cladophoropsis* and *Cladophora* (Ulvophyceae). It was also compared with simple Rhodophyta (e.g., *Rhodoehorton*), but Butterfield et al. (1994)[48] considered that the varied cell length in *Proterocladus* is similar to that in modern *Cladophoropsis*, whereas its branching pattern and septal structure are similar to those in *Cladophora*. Butterfield (2009)[49] noted "the generally large cell size (up to 50 m wide and 1000 m long) strongly implies a multinucleate grade of organization" which is consistent with early branching Ulvophyceae. These remains occur in the sediments of the Svanbergfjellet Formation, NE Spitzbergen, which is considered to be 700-750 Ma. *Proterocladus* spp. were used to calibrate the stem node of Cladophorales with a lognormal prior distribution with mean = 3.2741, standard deviation = 0.75, and offset = 700.

Node D: Sphaeropleales stem node calibrated with *Palaeastrum dyptocranum* Butterfield, Knoll & Swett, from the late Riphean (Neoproterozoic)[48]. *Palaeastrum* are spheroidal to ellipsoidal cells with prominent intercellular attachment circular discs with a reinforced rim, which form monostromatic colonies. "The intercellular attachment discs are not simply the product of cell-cell contact but are fully differentiated structures involved in the maintenance of colony structure."[48]. *Palaeastrum* was described as forming monostromatic colonies from 15 to >100 cells, similar to extant *Pediastrum, Hydrodictyon* and *Coelastrum* in grade of multicellular organization, and probably related to sphaeroplealean chlorophytes[48-49]. *Palaeastrum* was found in the sediments of the Svanbergfjellet Formation, NE Spitzbergen, which is considered to be 700-750 Ma. *Palaeastrum dyptocranum* was used to calibrate the stem node of Sphaeropleales with a lognormal prior distribution with mean = 3.2741, standard deviation = 0.75, and offset = 700.

Node E: Chlorellales (Trebouxiophyceae) stem node calibrated with *Caryosphaeroides pristina* Schopf, from the Late Precambrian[50]. *Caryospaheroides pristina* are spheroidal cells that are solitary or forming small colonies with typical chlorococcoid appearance, similar to modern *Chlorococcum* or *Chlorella*[50]. Cells with a single, dense granular body were originally interpreted as a nucleus, but this interpretation was controversial[51]. The cell size (average of 13 µm) and presence of an internal body that was interpreted as a pyrenoid after TEM investigation is consistent with *Caryosphaeroides* being a green

alga[52-53]). *Caryosphaeroides pristina* was reported from the late Precambrian Bitter Springs Formation, central Australia, which is ca. 800-830 Ma. Age consistent with prior estimated divergence time for group[54]. *Caryosphaeroides pristina* was used to calibrate the stem node of Chlorellales with a lognormal prior distribution with mean = 3.6308, standard deviation = 0.75, and offset = 1000.

Node F: Embryophytes crown node bounded with a maximum age corresponding to the oldest stem embryophytes (Middle Ordovician, Dapingian[55]), and a minimum age corresponding to the oldest crown embryophytes (Upper Ordovician[56]). The maximum age of the embryophyte crown group was bounded by what possibly represent the oldest stem lineage embryophytes, corresponding to assemblages of sporopolleninn-coated monads, dyads and tetrads of cryptospores (spores that lack haptophytic features such as a trilete or monolete mark) with different ornamentations, from Dapingian (early Middle Ordovician) sediments from Argentina (eastern Gondwana[55]). Their relationship with embryophytes is demonstrated by their ability to produce sporopollenin, which unambiguously represents an attribute of this lineage[55, 57]. Their organization as monads, dyads or tetrads, instead as free monads resulting from the dissociation of meiotic tetrads, and the occasional presence of a thin envelope that is difficult to equate with a similar structure among extant plants[57], suggest that these cryptospores represent embryophyte stem relatives. The minimal age of the embryophyte crown node was bounded by plant fragments including sporangia containing spores, from Ordovician sediments from Oman[56]. Spore wall ultrastructure supports affinities with liverworts, hence, membership into crown group embryophytes[56, 58]. The embryophyte crown node was calibrated with a uniform distribution between 485 and 443.8, which correspond to the lower and upper bounds of the Ordovician.

Node G: Metzgeriales stem node calibrated with *Metzgeriothallus sharonae* VanAller Hernick, Landing & Bartowski, from the Middle Devonian (Givetian)[59]. *Metgeriothallus sharonae* is the earliest secure liverwort known in the fossil record. It is preserved as a carbonaceous film, and consists of a dorsiventral thallus ca. 32 mm long and 1.5-3.8 mm wide, with a median costa (midrib) and entire-margined wings. The unicellular ribbon-like cells that extend from beneath the costa appear to be typical marchantoid rhizoids. Similarly, the sporophyte capsule that opens with four valves indicates a liverwort[59]. The bifurcating simple thalloid habitus with rhizoids extending from beneath the costa, however, narrow the liverwort appearance to the classic simple thalloid liverworts such as *Metzgeria* or *Pellia*. *Metzgeriothallus sharonae* was found in Middle to Late Devonian Catskill Delta succession. *Metzgeriothallus sharonae* was used to calibrate the stem node of Metzgeriales with a lognormal prior distribution with mean = 2.6703, standard deviation = 0.75, and offset = 382.7.

Node H: Hypnales stem node calibrated with *Capimirinus riopretensis* Cortez Christiano de Souza, Ricardi Branco & León Vargas, from the Permian[60]. Dichotomous branching stems with spirally distributed erect-patent-leaves that harbor a well-delimited single costa[60] clearly indicate the fossil as a moss. The leave shape is ovate and slightly asymmetric[60]. The well-preserved sporophyte that is developed at the apex of a short side branch[60], which defines pleurocarpy, places the fossil at the stem node of the included pleurocarps after the split from the Orthotrichales (compare Bell et al. 2007)[61]. *Capimirinus riopretensis* was used to calibrate the stem node of Hypnales with a lognormal prior distribution with mean = 2.2531, standard deviation = 0.75, and offset = 252.17.

Node I: Lycophytes stem node (equal to tracheophytes crown node) calibrated with *Baragwanathia longifolia* Lang & Cookson, from the Upper Silurian (Ludlow)[62-63]. *Baragwanathia* has been identified as a member of Lycopodiophyta due to the presence of microphylls. Morphology-based phylogenetic analyses placed *Baragwanathia* on the stem lineage of the extant Lycopodiaceae and Selaginellaceae/Isoetaceae[64]. *Baragwanathia* was used to calibrate the stem group of Lycopodiophyta (equal to the crown group of tracheophytes) with a lognormal prior distribution with mean = 2.7704, standard deviation = 0.75, and offset = 423.

Node J. Isoletaceae-Selaginellaceae stem node (equal to lycophytes crown node), calibrated with *Leclercquia complexa* Banks, Bonamo & Grierson, from the Middle Devonian[65]. Morphology-based phylogenetic analyses by Kenrick and Crane (1997)[64] placed *Leclerquia* in Lycopsida, within a clade that includes the living Isoetaceae and Selaginellaceae, as well as several extinct lineages. *Leclerquia* was used to calibrate the stem node of Selaginellaceae plus Isoetaceae (equal to the crown group of Lycopodiophyta) with a lognormal prior distribution with mean = 2.6703, standard deviation = 0.75, and offset = 382.7.

Node K: Equisetales stem node calibrated with *Ibyka amphikoma* Skog & Banks, from the Middle Devonian (Givetian[66-68]). *Ibyka amphikoma* consists of vegetative and reproductive smooth axes that branch pseudomonopodially in three dimensions, and ovoid to pyriform sporangia clustered in terminal pairs. Based on morphology-based phylogenetic analyses, *Ibyka* was identified as a stem relative of sphenophytes[64], and represents the oldest member of monilophytes. *Ibyka* was used to constrain the stem node of Equisetales, which is equal to the divergence between Equisetales and Polypodiales, with a lognormal prior distribution with mean = 2.6703, standard deviation = 0.75, and offset = 382.7.

Node L: Osmundaceae stem node (equal to leptosporangiate ferns crown node) calibrated with *Thamnopteris schlechtendalii* Kidston & Gwynne-Vaughan, from the Upper Permian[69]. *Thamnopteris schlechtendalii* is a structurally preserved arborescent stem with protostele anatomy, surrounded by a mantle of adventitious roots, similar to that in Osmundaceae[69]. *Thamnopteris schlechtendalii* was used to calibrate the stem node of Osmundaceae, corresponding to the crown node of leptosporangiate ferns, with a lognormal prior distribution with mean = 2.2531, standard deviation = 0.75, and offset = 252.17.

Node M: *Gleichenia* stem node calibrated with *Gleichenia chaloneri* Herendeen & Skog, from the early Albian[70]. Herendeen & Skog (2009)[70] found *G. chaloneri* to be the sister to *Gleichenia glauca*; hence, the fossil could calibrate stem node *Gleichenia*. However, Schuettpelz & Pryer (2009)[71] did not do this, because they considered that presently, *Gleichenia* is delimited differently. They used *G. chaloneri* to calibrate a subclade within Gleicheniaceae consisting of *Gleichenia*, *Sticherus* and *Stromatopteris*. However, this clade is represented in our tree only by *Gleichenia. Gleichenia chalonerii* was used to calibrate the stem node of *Gleichenia*, with a lognormal prior distribution with mean = 1.3332, standard deviation = 0.75, and offset = 100.5.

Node N: *Lygodium* stem node calibrated with *Stachypteris spicans* Pomel from the Middle Jurassic (Bajocian[72]). *Stachypteris spicans* were re-studied and described as trilete spores with "rounded-

=triangular in equatorial outline: distal surface convex in equatorial view, proximal surface rather flattened: laesurae distinct, reaching almost to the equator, sometimes bordered by an inconspicuous margo: exine thick … distal surface sculptured with muri and irregularly shaped pits, at the bottom of the pits granula visible; proximal surface finely granulate …"[72]. Based on morphological comparisons, *Stachypteris spicans* was considered most closely similar to reticulate spores of *Lygodium*[72]. *Stachypteris spicans* was used to calibrate the stem node of *Lygodium*, corresponding in our tree to the crown node of Schizaeaceae, with a lognormal prior distribution with mean = 1.8488, standard deviation = 0.75, and offset = 168.3.

Node O: Cyatheaceae stem node calibrated with *Cyathocaulis naktongensis* Ogura from the Upper Jurassic[73-74]. *Cyathocalulis naktongensis* is a tree fern stem covered with roots, showing spirally-arranged vascular traces, conspicuously curved meristeles enclosed in schlerenchymatous sheath, numerous medullary bundles mostly close to the center of the pith or near the leaf-gap. Leaf scars are elliptical or fusiform with numerous small bundles, arranged as in living Cyatheaceae[73]. A phylogenetic analysis of extant and fossil tree ferns based on stem anatomy placed *C. naktongensis* as stem member of a clade that includes *Alsophila*, *Cyathea*, *Hymenophyllopsis* and *Sphaeropteris*[74]. *Cyathocaulis naktongensis* was used to calibrate the stem node of Cyatheaceae, with a lognormal prior distribution with mean = 1.6998, standard deviation = 0.75, and offset = 145.

Node P: Pteridaceae stem node calibrated with "*Pteris* sp." from the Cenomanian[75]. "*Pteris* sp." are fragments of pinnate leaves with a thin, longitudinally striate rachis that forks distally at an acute angle. Pinnae are alternate, lanceolate, variably dissected, with 1-2 pairs of proximal pinnules. Pinnules are anadromous or catadromous, elliptical to rounded, reduced to lobes in the distal pinnae, with pinnate venation. Fertile pinnae have linear marginal coenosori[75]. Krassilov and Bachia (2000)[75] considered that pinnule morphology and position of the coenosori are typical of the extant genus *Pteris*. Schuettpelz and Pryer (2009)[71] considered this fossil to be related to Pteridaceae, but not necessarily to genus *Pteris*. "*Pteris* sp." was used to calibrate the stem node of Pteridaceae, with a lognormal prior distribution with mean = 1.2652, standard deviation = 0.75, and offset = 93.9.

Node Q: Spermatophytes stem node (equal to euphyllophytes crown node) calibrated with *Pertica quadrifaria* Kasper & Andrews and *P. varia* Granoff et al., from the Lower Devonian (Emsian[76-77]). *Pertica* are distally isotomous, pseudomonopodially branched axes with a decussate lateral branches resulting in a tetrastichious arrangement, and fertile ultimate appendages with terminally-borne clusters of erect sporangia[64, 78]. In morphology-based phylogenetic analyses, *Pertica* is placed within crown euphyllophytes, on lineage leading to seed plants[64]. *Pertica quadrifaria* was used to calibrate the stem node of spermatophytes (equal to the crown node of euphyllophytes) with a lognormal prior distribution with mean = 2.6976, standard deviation = 0.75, and offset = 393.3.

Node R: Cycads crown node calibrated with *Crossozamia* Pomel emend. Gao & Thomas, from the Lower Permian[79-80]. Four species of *Crossozamia* described from compression-impression specimens. The fossils are megasporophylls with fan-shaped to palmate lamina, distal margin divided into tapering segments, with a long and broad stalk, ovoid to oblong ovules attached laterally on each side of the stalk, with one or two ovules borne at proximal lateral margins of the lamina. Hermsen et al. (2006)[80]

included *Crossozamia* in a morphology-based phylogenetic analysis and found it to be sister to extant *Cycas*. Considering this phylogenetic result, *Crossozamia* indicates the differentiation of Cycadaceae and Zamiaceae, which corresponds to the cycadophyte crown node. *Crossozamia* spp. was used to calibrate the crown node of cycads with a lognormal prior distribution with mean = 2.3299, standard deviation = 0.75, and offset = 272.3.

Node S: Conifer stem node (equal to conifers = ginkgophytes crown node) calibrated with *Swillingtonia denticulata* Scott & Chaloner from the Westphalian B (Middle Pennsylvanian, Carboniferous[81-82]). *Swillingtonia denticulata* are small leaves and leafy shoots preserved as compressions and charcoal, including epidermal characters, and internal structure. Leaves are hyposotmatic with a denticulate margin, and the lower epidermis has two wide stomatal bands, often with subsidiary cells[81]. Epidermal, stomatal and anatomical features indicate the relationship of these remains to conifers. *Swillingtonia denticulata* was used to calibrate the stem node of conifers (including gnetophytes), with a lognormal prior distribution with mean = 2.4499, standard deviation = 0.75, and offset = 307.

Node T: *Picea* stem node calibrated with *Picea burtonii* Klymiuk & Stockey, and *Pinus* stem node calibrated with *Pinus mundayi* Falcon-Lang Mages & Collinson. The calibrations correspond to the divergence between *Pinus* and *Picea* in our tree, and both fossils are from the Valanginian[83-84]. *Picea burtonii* is an anatomically preserved seed cone with a combination of characters present only in *Picea*. A morphology-based phylogenetic analysis placed *Picea burtonii* as most closely related to *Picea* among extant genera[84]. *Pinus mundayi* are anatomically preserved long shoots with axial resin ducts with thin-walled epithelial cells in the secondary xylem and phloem; fenestriform or pinoid cross-field pits; and helically arranged short-shoots that pass through growth ring boundaries before distally diverging into two separate needle bases. It was interpreted as belonging to an evergreen two-needle pine[83]. The *Pinus-Picea* divergence was calibrated with a lognormal prior distribution with mean = 1.6126, standard deviation = 0.75, and offset = 132.9.

Node U: Gnetophytes stem node calibrated with *Dechellyia gormani* Ash, and *Maculostrobus clatratus* Ash, from the Upper Triassic[85-86]. *Dechellyia gormani* are sterile and fertile branches with leaves arranged in opposite and decussate pairs, and oval seeds attached to a lanceolate lamina[85]. *Masculostrobus clathratus* are cones with *Equisetosporites*-type pollen. Although these organs have not been found in connection, their combined features indicate an affinity with gnetophytes[86]. These fossils are here considered to be stem lineage representatives of gnetophytes, and are used to calibrate their stem node. The stem node of gnetophytes was calibrated with a lognormal prior distribution with mean = 2.0278, standard deviation = 0.75, and offset = 201.3.

Node V: *Ephedra* stem node (equal to gnetophyte crown node) calibrated with *Liaoxia chenii* Cao et S.Q. (emend. Rydin, Wu & Friis), and *Ephedra archaeorhytidosperma* Yang, Geng, Dilcher, Chen & Lott, both from the Early Cretaceous (approximately Barremian[87-88]). *Liaoxia chenii* corresponds to plants similar to *Ephedra* with erect striate stems distinct nodes, and opposite decussate branching. Leaves, when present, are linear. Reproductive cones are rounded to ovobate with opposite-decussate bracts, with ovoid to elliptic seeds in the axils of bracts[88]. *Ephedra archaeorhytidosperma* are fertile branches,

including female cones and seeds preserving morphologial and anatomical details, including the mycropilar tube, which can be assigned to Ephedraceae[87]. *Liaoxia chenii* and *Ephedra archaeorhytidosperma* are here considered as stem relatives of *Ephedra*, and used to calibrate the stem node of this genus, equivalent to the crown node of gnetophytes, with a lognormal prior distribution with mean = 1.5513, standard deviation = 0.75, and offset = 125.

Node W: Araucariaceae stem node (equal to the divergence between Araucariaceae and Podocarpaceae) calibrated with *Araucarites phillipsii* Harris and *Brachyphyllum mamillare* Harris and from the Middle Jurassic (Aalenian[89-90]). *Araucarites phillipsii* are ovulate cones with seeds fused to the bract for most of its length except at the tip, and covered by cone scale tissue as in extant Araucariaceae[90]. *Brachyphyllum mammilare* are leaves and attached pollen cones considered to belong to the same plant as *Araucarites phillipsii*[89-90]. Both taxa show attributes of Araucariaceae[90]. We follow Leslie et al. (2012)[90] in considering both as the earliest fossils that can be confidently assigned to Araucariaceae, and use them to calibrate the stem node of this family with a lognormal prior distribution with mean = 1.8606, standard deviation = 0.75, and offset = 170.3.

Node X: Taxaceae stem node calibrated with *Palaeotaxus rediviva* Florin, from the Lower Jurassic (Hettangian[91]). *Palaeotaxus rediviva* is known from vegetative remains and ovulate cones. Leslie et al. (2012)[90] considered *Palaeotaxus rediviva* similar to extant Taxaceae in having an axillary shoot with sterile scales that terminates in a single ovule. *Palaeotaxus rediviva* was here used to calibrate the stem node of Taxaceae with a lognormal prior distribution with mean = 2.0178, standard deviation = 0.75, and offset = 199.3.

Node Y: Angiospermae crown node calibrated with pollen grains with infratectal columellae and reticulate tectum, from the Valanginian-Hauterivian. The age of the angiosperm crown node was bounded uniformly between the age of the oldest fossils that can reliably be assigned to the group, and the oldest bound of the age of this node estimated in a diversification analysis based on fossil occurrences[92]. Pollen grains with infratectal columellae and perforate to reticulate tectum from late Valaginian and Hauterivian sediments[93-95]. The presence of a collumellate infratectal structure and the distribution of that attribute among early diverging angiosperm lineages indicates affinity with angiosperms[96]. One among several most parsimonious optimizations is that the columellar infratectum and perforate to reticulate tectum observed in the oldest angiosperm fossil pollen grains evolved within crown group angiosperms. If this reconstruction is correct, then the oldest known angiosperm fossils would be members of the angiosperm crown group[97-98]. We used the Valanginian-Hauterivian age of pollen grains with collumellate infratectum and perforate to reticulate tectum to determine the minimum bound of the confidence interval at 132.9 Ma. The maximum age of the inteval was obtained from the upper bound of the age of the angiosperms estimated with a hierarchical Bayesian model to estimate temporal dynamics of speciation and extinction based on fossil occurrences of angiosperm genera, and which jointly models diversification and preservation potential[92, 99]. The age of crown group angiosperms was estimated as having a Laplace distribution centered on 143.8 Ma, and with a 95% HPD between 151.8 and 133.0 Ma[92]. We used the oldest limit of the 95% HPD as the maximum bound of the uniform distribution to calibrate the angiosperm crown node. Therefore, the angiosperm crown node was assigned a uniform prior distribution between 132.9 and 151.8.

Node Z: Nymphaeaceae stem node calibrated with *Monetianthus mirus* Friis, Pedersen, von Balthazar, Grimm & Crane, from the late Aptian-early Albian[100-101]. *Monetianthus mirus* is known from a single three-dimensionally preserved flower. It is radially symmetrical, perigynous and apparently bisexual. Based on floral traits, an affinity with Nymphaeaceae was initially proposed[102], and subsequently confirmed by the type of placentation, ovule number and size, revealed by Synchrotron Radiation X-Ray Tomographic Microscopy SRXTM[100]. *Monetianthus mirus* is considered as a stem representative of Nymphaeaceae, and was used to calibrate the split between Nymphaeaceae and Cabombaceae, with a lognormal prior distribution with mean = 1.4144, standard deviation = 0.75, and offset = 109.

Node a: Schisandraceae stem node calibrated with *Anacostia* spp. Friis, Crane & Pedersen, from the late Aptian-early Albian[103]. A phylogenetic analysis conducted by Doyle *et al.* (2008)[104] placed a consensus of the four species of *Anacostia* described by Friis *et al.* (1997)[103] within the Austrobaileyales, as the sister taxon of *Schisandra* plus *Illicium*. *Anacostia* is here considered as a stem representative of Schisandraceae, and is used to calibrate the Schisandraceae stem group, which is equivalent to the Austrobaileyales crown node, with a lognormal prior distribution with mean = 1.4144, standard deviation = 0.75, and offset = 109.

Node b: Chloranthales crown node calibrated with the *Asteropollis* plant, from the late Barremian to the early Aptian[105-106]. The *Asteropollis* plant consists of epigynous trigonous pistillate flowers and fruits[105] with attached pollen with a star-shaped aperture, corresponding to *Asteropollis*[106], and staminate inflorescence and dispersed stamens. The flowers and pollen are very similar to extant *Hedyosmum*[105-106]. A phylogenetic analysis indicated the placement of the *Asteropollis* plant among extant species of *Hedyosmum*[107]. We consider the *Asteropollis* plant to represent a stem member of *Hedyosmum*, hence, it would calibrate the stem node of this genus, which is equivalent to the crown node of Chloranthaceae. Because our analysis included a single terminal for Chloranthales, the *Asteropollis* plant was used to calibrate the divergence between Chloranthales and Magnoliales with a lognormal prior distribution with mean = 1.5188, standard deviation = 0.75, and offset = 121.

Node c: Magnoliales crown calibrated with *Endressinia brasiliana* Mohr & Bernardes-de-Oliveira, from the late Aptian to early Albian[108]. Flowers of *Endressinia brasiliana* have an undifferentiated perianth, broad staminodes and ca. 20 independent plicate carpels. It shares features with nearly all families of Magnoliales, and the presence of broad staminodes and glands suggest a relationship with Eupomatiaceae[108]. *Endressinia* has an equally parsimonious position as sister to a clade that includes *Galbulimima*, *Degeneria*, *Eupomatia* and Annonaceae (Fig. 3 in Doyle and Endress, 2010[109]), or in all positions immediately below and within this clade (p. 12 in Doyle and Endress, 2010[109]). We here consider *Endressinia* as a crown group member of Magnoliales, and used it to calibrate this node with a lognormal prior distribution with mean = 1.4144, standard deviation = 0.75, and offset = 109.

Node d: Lauraceae stem node calibrated with *Potomacanthus lobatus* von Balthazar, Pedersen, Crane, Stampanoni & Friis, from the early-middle Albian[110]. *Potomacanthus* are trimerous flowers that include stamens with bisporangiate anthers with valvate dehiscence, with affinities to Lauraceae[110]. *Potomacanthus* is considered a stem representative of Lauraceae, and was used to calibrate the stem

node of this family, with a lognormal prior distribution with mean = 1.3741, standard deviation = 0.75, and offset = 104.7.

Node e: Winteraceae stem node calibrated with *Walkeripollis gabonensis* Doyle, Ward & Hotton, from the late Barremian[109, 111]. *Walkeripollis gabonensis* are dispersed pollen tetrads with a fine sculpture and tectal connections among the component monads. Morphology-based phylogenetic analyses found that the most parsimonious position of *Walkeripollis gabonensis* is as sister to Winteraceae[109]. Based on this result, *Walkeripollis* is used to calibrate the Winteraceae stem group (equal to crown group Canellales), with a lognormal prior distribution with mean = 1.5513, standard deviation = 0.75, and offset = 125.

Node f: Araceae crown node calibrated with *Mayoa portugallica* Friis, Pedersen & Crane, from the late Barremian-early Aptian[112]. *Mayoa portugallica* are striate and inaperturate pollen grains similar in detail to those of genera of Monsteroideae (Araceae). The assignment of *Mayoa* to tribe Spathiphylleae [Monsteroideae, Araceae[112]] would allow it to calibrate the crown node of Araceae, but because of the sparse sampling in this analysis, it is used to calibrate the stem node of Alismatales with a lognormal prior distribution with mean = 1.5188, standard deviation = 0.75, and offset = 121.

Node g: Arecaceae stem node calibrated with leaves assigned to *Sabal magothiensis* Berry and stems assigned to *Palmoxylon cliffwoodensis* Berry, from the Santonian[113]. Leaves, stems and pollen grains from the Magothy Formation were assigned to Arecaceae[113]. These remains exhibit distinctive attributes of Arecaceae, but can belong to the stem group or the crown group of the family. They were used to calibrate the Arecaceae stem group, with a lognormal prior distribution with mean = 1.1491, standard deviation = 0.75, and offset = 83.6.

Node h: Eudicotyledoneae crown node calibrated with tricolpate pollen grains from the Barremian-Aptian. Tricolpate pollen grains from mid- to late Barremian sediments, and from close to the Barremian-Aptian boundary[114-116]. Tricolpate pollen grains unequivocally characterize eudicots (Eudicotyledoneae). The records correspond to isolated tricolpate pollen grains from the middle Atherfield Wealden Bed 35, corresponding to the mid- to late Barremian in the Early Cretaceous succession in southern and eastern England[114], and to aff. *Tricolpites micromurus* and aff. *Tricolpites crassimurus* from Pollen Zone C-VII of the Cocobeach sequence, Gabon, corresponding closely to the Barremian-Aptian boundary[115-116]. Because it is very likely that tricoplate pollen evolved along the stem lineage of eudicots, the fossil tricolpate pollen grains are used to calibrate the stem node of Eudicotyledoneae (equivalent to the split with Ceratophyllales) with a lognormal prior distribution with mean = 1.5513, standard deviation = 0.75, and offset = 125.

Node i: Ranunculales crown node calibrated with *Teixeiraea lusitanica* von Balthazar, Pedersen & Friis, from the late Aptian[117]. Flowers assigned to *Teixeiraea lusitanica* are actinomorphic, with helical phyllotaxy, with a moderately differentiated perianth, and stamens of two sizes containing tricolpate pollen with a perforate tectum. No carpels were observed; hence, it is interpreted as a unisexual staminate flower. Von Balthazar et al. (2005)[117] considered it to be related to members of Ranunculales, but a possible affinity with Hamamelidaceae and Daphniphyllaceae (Saxifragales) and

Berberidopsidaceae (Berberidopsidales) within Pentapetalae was also considered. *Teixeiraea lusitanica* was used to calibrate the crown node of Ranunculales with a lognormal prior distribution with mean = 1.4504, standard deviation = 0.75, and offset = 113.

Node j: Mensipermaceae stem node calibrated with *Prototinomiscium testudinarum* Knobloch & Mai and *P. vangerowii* Knobloch & Mai from the Maastrichtian sediments[118]. *Prototinomiscium* spp. are endocarps with features corresponding to Menispermaceae. These fossils could belong to the stem lineage or to the crown group of Menispermaceae, and were used to calibrate the Menispermaceae stem node, with a lognormal prior distribution with mean = 0.9127, standard deviation = 0.75, and offset = 66.

Node k: Platanaceae stem node calibrated with leaves of *Sapindopsis variabilis* Fontaine; staminate inflorescences and flowers assigned to *Aquia brookensis* Crane, Pedersen, Friis & Drinnan; and pistillate inflorescences and flowers assigned to *Platanocarpus brookensis* Crane, Pedersen, Friis & Drinnan, from the early-middle Albian[119]. These detached organs were reconstructed by Crane et al. (1993)[119] as belonging to a single plant taxon, referred to as the *Sapindopsis* plant. Morphology-based phylogenetic analyses placed the reconstructed *Sapindopsis* plant as sister to extant *Platanus* (Platanaceae). The reconstructed plant was considered as a stem representative of Platanaceae, and used to calibrate the Platanaceae stem node with a lognormal prior distribution with mean = 1.3741, standard deviation = 0.75, and offset = 104.7.

Node l: Pentapetalae crown node calibrated with the “Rose Creek Flower”, from the latest Albian[120]. The Rose Creek Flower is the oldest fossil showing the distictive floral ground plan of Pentapetalae, including a two-whorled perianth differentiated into distinct calyx and corolla, pentamerous merosity, and radial alternation between the organs of concentric whorls. Stamens are clearly differentiated into filament and anther, and the gynoecium is syncarpous and superior[120]. While its floral ground plan confidently documents an affinity with Pentapetalae, a relationship with any major clade within Pentapetalae is equivocal[101]. We used the Rose Creek Flower to calibrate the crown node of Pentapetalae with a lognormal prior distribution with mean = 1.3332, standard deviation = 0.75, and offset = 100.5.

Node m: Ericales crown node calibrated with *Paleoenkianthus sayrevillensis* Nixon & Crepet, from the Turonian[121]. *Paleoenkianthus sayrevillensis* are flowers with five sepals, five petals, eight stamens and four carpels[121]. The combination of its floral traits and anatomical features suggested an affinity with several families of Ericales, especially Ericaceae[121]. This fossil was considered to be related to Clethraceae, Cyrillaceae or Ericaceae, and was used to calibrate the crown node of Ericales with lognormal prior distribution with mean = 1.2206, standard deviation = 0.75, and offset = 89.8.

Node n: Brassicales stem node calibrated with *Dressiantha bicarpelata* Gandolfo, Nixon & Crepet, from the Turonian[122]. The flowers assigned to *Dressiantha bicarpelata* exhibit a unique combination of attributes relative to extant taxa, including a gynophore, a 2 + 2 arrangement of the sepals, unequal petals, monothecal anthers, and a bicarpellate gynoecium”[122]. Among extant taxa, this combination occurs only among Capparaceae, Brassicaceae, and related families within Brassicales.

Dressiantha bicarpelata was used to calibrate the stem node of Brassicales, with lognormal prior distribution with mean = 1.2206, standard deviation = 0.75, and offset = 89.8.

Node o: Fagales crown node calibrated with pollen grains of the Normapolles complex, from the middle Cenomanian[123-125]. Pollen grains of the Normapolles complex are oblate, triangular in polar view, with protruding, complex apertures and tectate, slighlty sculptured pollen wall[126]. These morphological attributes suggested a general affinity with Fagales, which became very strongly supported by the finding of Normapolles grains in situ structurally preserved flowers with affinities to Juglandaceae/Myricaceae, or Betulaceae, all within the core Fagales. Therefore, Normapolles pollen grains are used to calibrate the crown group of Fagales with a lognormal prior distribution with mean = 1.2998, standard deviation = 0.75, and offset = 97.2.

Supplementary References

1. Soltis, P. S., Soltis, D. E., Savolainen, V., Crane, P. R. & Barraclough, T.G. Rate heterogeneity among lineages of tracheophytes: integration of molecular and fossil data and evidence from molecular living fossils. *Proc. Natl Acad. Sci. USA* **99**, 4430–4435 (2002).

2. Sanderson, M. J. Molecular data from 27 proteins do not support a Precambrian origin of land plants. *Am. J. Bot.* **90**, 954–956 (2003).

3. Magallón, S. & Sanderson, M. J. Angiosperm divergence times: the effect of genes, codon positions, and time constraints. *Evolution* **59**, 1653–1670 (2005).

4. Hackett, J. D., Yoon, H. S., Reyes-Prieto, A., Rümmele, S. E. & Bhattacharya, D. Phylogenetic analysis supports the monophyly of cryptophytes and haptophytes and the association of *Rhizaria* with Chromalveolates. *Mol. Phylogenet. Evol.* **24**, 1702–1713 (2007).

5. Bell, C. D., Soltis, D. E. & Soltis, P. S. The age and diversification of the angiosperms re-revisited. *Am. J. Bot.* **97**, 1296–1303 (2010).

6. Magallón, S. Using fossils to break long branches in molecular dating: a comparison of relaxed clocks applied to the origin of angiosperms. *Syst. Biol.* **59**, 384–399 (2010).

7. Smith, S. A., Beaulieu, J. M. & Donoghue, M. J. An uncorrelated relaxed-clock analysis suggests an earlier origin for flowering plants. *Proc. Natl Acad. Sci. USA* **107**, 5897–5902 (2010).

8. Clarke, J. T., Warnock, R. C. M. & Donoghue, P. C. J. Establishing a time-scale for plant evolution. *New Phytol.* **192**, 266–301 (2011).

9. Magallón, S., Hilu, K. W. & Quandt, D. Land plant evolutionary timeline: Gene effects are secondary to fossil constraints in relaxed clock estimation of age and substitution rates. *Am. J. Bot.* **100**, 556–573 (2013).

10. Berbee, M. L. & Taylor, J. W. Dating the evolutionary radiations of the true fungi. *Can. J. Bot.* **71**,1114–1127 (1993).

11. Heckman, D. S. *et al.* Molecular evidence for the early colonization of land by fungi and plants. *Science* **293**, 1129–1133 (2001).

12. Douzery, E. J. P., Snell, E. A., Bapteste, E., Delsuc, F. & Philippe H. The timing of eukaryotic evolution: Does a relaxed molecular clock reconcile proteins and fossils? *Proc. Natl Acad. Sci. USA* **101**, 15386–15391 (2004).

13. Padovan, A. C. B, Sanson, G. F. O., Brunstein, A. & Briones, M. R. S. Fungi evolution revisited: Application of the penalized likelihood method to a Bayesian fungal phylogeny provides a new perspective on phylogenetic relationships and divergence dates of Ascomycota groups. *J. Mol. Evol.* **60**, 726–735 (2005).

14. Taylor, J. W. & Berbee, M. L. Dating divergences in the Fungal Tree of Life: review and new analyses. *Mycologia* **98**, 838–849 (2006).

15. Gueidan, C., Ruibal, C., de Hoog, G. S. & Schneider, H. Rock-inhabiting fungi originated during periods of dry climate in the late Devonian and middle Triassic. *Fungal Biol.* **115**, 987–996 (2011).

16. Floudas, D. *et al.* The Paleozoic origin of enzymatic lignin decomposition reconstructed from 31 fungal genomes. *Science* **336**, 1715–1719 (2012).17. Yang, Z. & Rannala, B. Bayesian estimation of species divergence times under a molecular clock using multiple fossil calibrations with soft bounds. *Syst. Biol.* **23**, 212–226 (2006).

18. James, T. Y. *et al.* Reconstructing the early evolution of the fungi using a six-gene phylogeny. *Nature* **443**, 818–822 (2006).

19. Drummond, A. J. & Rambaut, A. BEAST: Bayesian evolutionary analysis by sampling trees. *BMC Evol. Biol.* **7**, 214 (2007).

20. Kirk, P. M., Cannon, P. F., Minter, D. W. & Stalpers, J. A. *Dictionary of the Fungi* (CAB International, Wallingford, 2008).

21. Alfaro, M. E. *et al.* Nine exceptional radiations plus high turnover explain species diversity in jawed vertebrates. *Proc. Natl Acad. Sci. USA* **106**, 13410–13414 (2009).

22. Lewis, L. A. & McCourt, R. M. Green algae and the origin of land plants. *Am. J. Bot.* **91**, 1535–1556 (2004).

23. Shaw, J. & Renzaglia, K. Phylogeny and diversification of bryophytes. *Am. J. Bot.* **91**, 1557–1581 (2004).

24. Mabberley, D. J. *Mabberley's Plant Book. A Portable Dictionary of Plants, Their Classification and Uses* 3rd edn (Cambridge University Press, 2008).

25. Smith, A. R. *et al.* A classification for extant ferns. *Taxon* **55**, 705–731 (2006).

26. Stevens, P. F. Angiosperm Phylogeny Website. http://www.mobot.org/MOBOT/research/APweb/ (2012).

27. Kar, R. K., Sharma, N. & Kar, R. Occurrence of fossil fungi in dinosaur dung and its implication on food habit. *Curr. Sci.* **87**, 1053–1056 (2004).

28. Taylor, T. N., Hass, H., Kerp, H., Krings, M. & Hanlin, R. T. 2005. Perithecial ascomycetes from the 400 million year old Rhynie chert: an example of ancestral polymorphism. *Mycologia* **97**, 269–285 (2005a).

29. Rikkinen, J. & Poinar, G. Fossilised *Anzia* (*Lecanorales*, lichen-forming *Ascomycota*) from European Tertiary amber. *Mycol. Res.* **106**, 984–990 (2002).

30. Dörfelt, H. & Schmidt, A. R. A fossil *Aspergillus* from Baltic amber. *Mycol. Res.* **109**, 956–960 (2005).

31. Rikkinen, J., Dörfelt, H., Schmidt, A. R., & Wunderlich, J. Sooty moulds from European Tertiary amber, with notes on the systematic position of *Rosaria* (‘*Cyanobacteria’*). *Mycol. Res.* **107**, 251–256 (2003).

32. Poinar, G. O. *Life in Amber* (Stanford University Press, Stanford, 1992).

33. Wunderlich, J. Zur Konservierung von Bernstein-Einschlüssen und über den `Bitterfelder Bernstein'. *Neue Entomol. Nachr.* **4**, 11–13 (1983).

34. Poinar, G. O. & Poinar, R. *The Amber Forest: A Reconstruction of a Vanished World* (Princeton University Press, Princeton, 1999).

35. Poinar, G. O. & Singer, R. Upper Eocene gilled mushroom from the Dominican Republic. *Science* **248**, 1099–1101 (1990).

36. Hibbett, D. S., Grimaldi, D. & Donoghue, M. J. Fossil mushrooms from Miocene and Cretaceous ambers and the evolution of Homobasidiomycetes. *Am. J. Bot.* **84**, 981–991 (1997).

37. LePage, B. A., Currah, R. S., Stockey, R. A. & Rothwell, G. W. Fossil ectomycorrhizae from the Middle Eocene. *Am. J. Bot.* **84**, 410–412 (1997).

38. Smith, S. Y., Currah, R. S. & Stockey, R. A. Cretaceous and Eocene poroid hymenophores from Vancouver Island, British Columbia. *Mycologia* **96**, 180–186 (2004).

39. Taylor, T. N., Remy, W., Hass, H. & Kerp, H. Fossil arbuscular mycorrhizae from the Early Devonian. *Mycologia* **87**, 560–573 (1995).

40. Taylor, T. N., Krings, M., Klavins, S. D. & Taylor, E. L. *Protoascon missouriensis*, a complex fossil microfungus revisited. *Mycologia* **97**, 725–729 (2005b).

41. Taylor, T. N., Hass, H. & Remy, W. Devonian Fungi: Interactions with green alga *Palaeonitella*. *Mycologia* **84**, 901–910 (1992).

42. Parfrey, L. W., Lahr, D. J. G., Knoll, A. H., & Katz, L. A. Estimating the timing of early eukaryotic diversification with multigene molecular clocks. *Proc. Natl Acad. Sci. USA* **108**, 13624–13629 (2011).

43. Butterfield, N. J. *Bangiomorpha pubescens* n. gen., n. sp.: Implications for the evolution of sex, multicellularity, and the Mesoproterozoic/Neoproterozoic radiation of eukaryotes. *Paleobiology* **26**, 386–404 (2000).

44. Knoll, A. H. The multiple origins of complex multicellularity. Annual Review of Earth and Planetary Sciences **39**, 217–239 (2011).

45. Yang, E. C., Boo, S. M., Bhattacharya, D., Saunders, G. W., Knoll, A. H., Fredericq, S., Graf, L. & Yoon, H. S. Divergence time estimates and the evolution of major lineages in the florideophyte red algae. *Sci. Rep.-UK* **6**, 21361 (2015).

46. Knoll, A. H., Wo, S. M., Bhattacharya, D., Saunders, G. W., Knoll, A. H., Fredericq, S., Graf, L. & Yoon, H. S. Divergence time estimates and the evolution*Palaios* **28**, 453–470 (2013).

47. Samuelsson, J. *et al.* Organic walled microfossils from Proterozoic Thule Supergroup, Northwestern Greenland. *Precambrian Res.* **96**, 1–23 (1999).
48. Butterfield, N. J., Knoll, A. H. & Swett, K. Paleobiology from the Neoproterozoic Svanbergfjellet Formation, Spitsbergen. *Fossils & Strata* **34**, 1–84 (1994.).
49. Butterfield, N. J. Modes of pre-Ediacaran multicellularity. *Precambrian Res.* **173**, 201–211 (2009).
50. Schopf, J. W. Microflora of the Bitter Springs Formation, Late Precambrian, central Australia. *J. Palaeontol.* **42**, 651–688 (1968).
51. Knoll, A. H. & Barghoorn, E. S. Precambrian eukaryotic organisms: a reassessment of the evidence. *Science* **190**, 52–54 (1975).
52. Oehler, D. Z. Pyrenoid-like Structures in Late Precambrian Algae from the Bitter Springs Formation of Australia. *J. Paleontol.* **51**, 885–901 (1977).
53. Knoll, A. H. in *Paleobotany, Paleoecology, and Evolution* (ed. Niklas, K.) Vol. I, 17–54 (Praeger, New York, 1981).
54. Herron, M. D., Hackett, J. D., Aylward, F. O. & Michod, R. E. Triassic origin and early radiation of multicellular volvocine algae. *Proc. Natl Acad. Sci. USA* **106**, 3254–3258 (2009).
55. Rubinstein, C. V., Gerrienne, P., de la Puente, G. S., Astini, R. A. & Steemans, P. Early Middle Ordovician evidence for land plants in Argentina (eastern Gondwana). *New Phytol.* **188**, 365–369 (2010).
56. Wellman, C. H., Osterloff, P. L. & Mohiuddin, U. Fragments of the earliest land plants. *Nature* **425**, 282–285 (2003).
57. Wellman, C. H. The invasion of the land by plants: when and where? *New Phytol.* **188**, 306–309 (2010).
58. Taylor, W.A. Spores in earliest land plants. *Nature* **373**, 391–392 (1995).
59. VanAller Hernick, E. L., Landing, E. & Bartowski, K. E. Earth's oldest liverworts - *Metzgeriothallus sharonae* sp. nov. from the Middle Devonian (Givetian) of eastern New York, U.S.A. *Rev. Palaeobot. Palyo.* **148**, 154–162 (2008).
60. Cortez Christiano de Souza, I., Ricardi Branco, F. S. & León Vargas, Y. Permian bryophytes of western Gondwanaland from the Paraná Basin in Brazil. *Palaeontology* **55**, 229–241 (2012).
61. Bell, N., Quandt. D., O'Brien, T. J. & Newton, A. Taxonomy and phylogeny in the earliest diverging pleurocarps: square holes and bifurcating pegs. *Bryologist* **110**, 204–231 (2007).
62. Garrat, M. J. & Rickards, R. B. Pridoli (Silurian) Graptolites in association with Baragwanathia (Lycophytina). *Bull. Geol. Soc. Den.* **35**, 135–139 (1987).
63. Hueber, F. M. Thoughts on the early lycopsids and zosterophylls. *Ann. Mo. Bot. Gard.* **79**, 474–499 (1992).
64. Kenrick, P. & Crane, P. R. *The Origin and Early Diversification of Land Plants - A Cladisitc Study* (Smithsonian Institution Press, Washington, 1997).

65. Fairon-Demaret, M. Nouveaux specimens du genre *Leclercqia* Banks, H. P., Bonamo, P. M. & Grierson, J. D. 1972 du Givétien (?) du Queensland (Australie). *Bull. Inst. R. Sci. Nat. Belg. Sci. Terr.* **50**, 1–4 (1974).

66. Skog, J. E. & Banks, H. P. *Ibyka amphikoma*, gen. et sp. n., a new protoarticulate precursor from the late Middle Devonian of New York State. *Am. J. Bot.* **60**, 366–380 (1973).

67. Banks, H. P. in *Biostratigraphy of Fossil Plants: Successional and Paleoecological Analyses* (eds Dilcher, D. L. & Taylor, T. N.) 1–24 (Dowden, Hutchinson and Ross, Stroudsburg, 1980).

68. Stein, W. E. *Iridopteris eriensis* from the Middle Devonian of North America, with systematics of apparently related taxa. *Bot. Gaz.* **143**, 401–416 (1982).

69. Miller, C. N., Jr. Evolution of the fern family Osmundaceae based on anatomical studies. *Contrib. Mus. Paleontol. Univ. Mich.* **23**, 105–169 (1971).

70. Herendeen ,P. S. & Skog, J. E. *Gleichenia chaloneri*—a new fossil fern from the Lower Cretaceous (Albian) of England. *Int. J. Plant Sci.* **159**, 870–879 (1998).

71. Schuettpelz, E. & Pryer, K. M. Evidence for a Cenozoic radiation in ferns in an angiosperm-dominated canopy. *Proc. Natl Acad. Sci. USA* **106**, 11200–11205 (2009.).

72. van Konijnenburg-van Cittert, J. H. A. Schizaeaceous spores in situ from the Jurassic of Yorkshire, England. *Rev. Palaeobot. Palyo.* **33**, 169–81 (1981).

73. Ogura, Y. On the structure and affinities of some fossil tree-ferns from Japan. *Jour. Fac. Sci. Imp. Univ. Tokyo Sect. III, Botany*, **1**, 351–380 (1927).

74. Lantz, T. C., Rothwell, G. W. & Stockey, R. A. *Conantiopteris schuchmanii*, gen. et sp. nov., and the role of fossils in resolving the phylogeny of Cyatheaceae s.l. *J. Plant. Res.* **112**, 361–381 (1999).

75. Krassilov, V. & Bacchia, F. Cenomanian florule of Nammoura, Lebanon. *Cretaceous Res.* **21**, 785–799 (2000).

76. Kasper, A. E. & Andrews, N. H. *Pertica*, a new genus of Devonian plants from northern Maine. *Am. J. Bot.* **59**, 897–911 (1972).

77. Granoff, J. A., Gensel, P. G & Andrews, N. H. A new species of *Pertica* from the Devonian of eastern Canada. *Palaeontographica Abt. B* **155**, 119–128 (1976).

78. Banks, H. P. Reclassification of Psilophyta. *Taxon* **24**, 401–413 (1975).

79. Gao, Z. & Thomas, B. A. A review of fossil cycad megasporophylls, with new evidence of *Crossozamia* Pomel and its associated leaves from the Lower Permian of Taiyuan, China. *Rev. Palaeobot. Palyno.* **60**, 205–223 (1989).

80. Hermsen, E. J., Taylor, T. N., Taylor, E. L. & Stevenson, D. W. Cataphylls of the Middle Triassic cycad *Antarcticycas schopfii* and new insights into cycad evolution. *Am. J. Bot.* **93**, 724–738 (2006).

81. Scott, A. The earliest conifer. *Nature* **251**, 707–708 (1974).

82. Scott, A. C. & Chaloner, W. G. The earliest fossil conifer from the Westphalian B of Yorkshire. *Proc. R. Soc. Lond. B* **220**, 163–182 (1983).

83. Falcon-Lang, H., Mages, V. & Collinson, M. The oldest *Pinus* and its preservation by fire. *Geology* **44**, 303–306 (2016).

84. Klymiuk, A. A. & Stockey, R. A. A Lower Cretaceous (Valanginian) seed cone provides the earliest fossil record for *Picea* (Pinaceae). *Am. J. Bot.* **99**, 1069–1082 (2012).

85. Ash, S. R. Late Triassic plants from the Chinle Formation in northeastern Arizona. *Palaeontology* **15**, 598–618 (1972).

86. Crane, P. R. The fossil history of the Gnetales. *Int. J. Plant Sci.* **157** (suppl.), S50–S57 (1996).

87. Yang, Y., Geng, B.-Y., Dilcher, D. L., Chen, Z.-D. & Lott, T. A. Morphology and affinities of an Early Cretaceous *Ephedra* (Ephedraceae) from China. *Am. J. Bot.* **92**, 231–241 (2005).

88. Rydin, C., Wu, S. Q. & Friis, E. M. *Liaoxia* Cao et S.Q. (Gnetales): ephedroids from the Early Cretaceous Yixian Formation in Liaoning , northeastern China. *Plant Syst. Evol.* **262**, 239–265 (2006).

89. Harris, T. M. *The Yorkshire Jurassic Flora. V. Coniferales* (British Museum of Natural History, London, 1920).

90. Leslie, A. B., Beaulieu, J. M., Rai, H. S., Crane, P. R., Donoghue, M. J. & Mathews, S. Hemisphere-scale differences ion conifer evolutionary dynamics. *Proc. Natl Acad. Sci. USA* **109**, 16217–16221 (2012).

91. Florin R. On Jurassic taxads and conifers from north-western Europe and Eastern Greenland. *Acta Horti Bergiani* **17**, 257-402 (1958).

92. Silvestro, D., Cascales-Miñana, B., Bacon, C. D. & Antonelli, A. Revisiting the origin and diversification of vascular plants through a comprehensive Bayesian analysis of the fossil record. *New Phytol.* **207**, 425–436 (2015).

93. Brenner, G. J. in *Flowering Plant Origin, Evolution and Phylogeny* (eds Taylor, D. W. & Hickey, L. J.) 91–115 (Chapman and Hall, New York, 1996).

94. Hughes, N. F. & McDougall, A. B. Records of angiospermid pollen entry into the english early cretaceous succession. *Rev. Palaeobot. Palynol.* **50**, 255–272 (1987).

95. Hughes, N. F., McDougall, A. B. & Chapman, J. L. Exceptional new record of Cretaceous Hauterivian angiospermid pollen from southern England. *J. Micropalaeont.* **10**, 75–82 (1991).

96. Doyle, J. A. Evolutionary significance of granular exine structure in the light of phylogenetic analyses. *Rev. Palaeobot. Palyno.* **156**, 198–210 (2009).

97. Doyle, J. A. Early evolution of angiosperm pollen as inferred from molecular and morphological phylogenetic analyses. *Grana* **44**, 227–251 (2005).

98. Doyle, J. A. Molecular and fossil evidence on the origin of angiosperms. *Annu. Rev. Earth Planet. Sci.* **40**, 301–326 (2012).

99. Silvestro, D., Schnitzler, J., Liow, L. H., Antonelli, A. & Salamin, N. Bayesian estimation of speciation and extinction from incomplete fossil occurrence data. *Syst. Biol.* **63**, 349–367 (2014).

100. Friis, E. M., Pedersen, K. R., von Balthazar, M., Grimm, G. W. & Crane, P. R. *Monetianthus mirus* gen. et sp. nov., a Nymphalean flower from the Early Cretaceous of Portugal. *Int. J. Plant Sci.* **170**, 1086–1101 (2009).

101. Friis, E. M., Crane, P. R. & Pedersen, K. R. *Early Flowers and Angiosperm Evolution* (Cambridge University Press, Cambridge, 2011).

102. Friis, E. M., Pedersen, K. R. & Crane, P. R. Fossil evidence of water lilies (Nymphaeales) in the Early Cretaceous. *Nature* **410**, 357–360 (2001).

103. Friis, E., Crane, P. & Pedersen, K. *Anacostia*, a new basal angiosperm from the Early Cretaceous of North America and Portugal with trichotomocolpate/ monocolpate pollen. *Grana* **36**, 225–244 (1997).

104. Doyle, J. A., Endress, P. K. & Upchurch, G. R. Early cretaceous monocots: a phylogenetic evaluation. *Acta Musei Nationalis Pragae, B Hist. Naturalis* **64**, 59–87 (2008).

105. Friis, E., Pedersen, K. & Crane, P. Angiosperm floral structures from the Early Cretaceous of Portugal. *Plant Syst. Evol.* **8**, 31–49 (1994).

106. Friis, E., Pedersen, K. & Crane, P. Early angiosperm diversification: the diversity of pollen associated with angiosperm reproductive structures in Early Cretaceous floras from Portugal. *Ann. Mo Bot. Gard.* **86**, 259–296 (1999).

107. Eklund, H., Doyle, J. & Herendeen, P. Morphological phylogenetic analysis of living and fossil Chloranthaceae. *Int. J. Plant Sci.* **165**, 107–151 (2004).

108. Mohr, B. & Bernardes-de-Oliveira, M. *Endressinia brasiliana*, a magnolialean angiosperm from the lower Cretaceous Crato Formation (Brazil). *Int. J. Plant Sci.* **165**, 1121–1133 (2004).

109. Doyle, J. A. & Endress, P. K. Integrating Early Cretaceous fossils into the phylogeny of living angiosperms: Magnoliidae and eudicots. *J. Syst. Evol.* **48**, 1–35 (2010).

110. von Balthazar, M., Pedersen, K. R., Crane, P. R., Stampanoni, M. & Friis, E. M. *Potomacanthus lobatus* gen. et sp. nov., a new flower of probable Lauraceae from the Early Cretaceous (Early to Middle Albian) of eastern North America. *Am. J. Bot.* **94**, 2041–2053 (2007).

111. Doyle, J. A., Hotton, C. L. & Ward, J. V. Early Cretaceous tetrads, zonasulcate pollen and Winteraceae. I. Taxonomy, morphology, and ultrastructure. *Am. J. Bot.* **77**, 1544–1557 (1990a).

112. Friis, E. M., Pedersen, K. R. & Crane, P. R. Araceae from the Early Cretaceous of Portugal: Evidence on the emergence of monocotyledons. *Proc. Natl Acad. Sci. USA* **101**, 16565–16570 (2004).

113. Daghlian, C. P. A review of the fossil record of monocotyledons. *Bot. Rev.* **47**, 517–555 (1981).

114. Hughes, N. F. & McDougall, A. B. Barremian-Aptian angiospermoid pollen records from southern England. *Rev. Palaeob. Palyno.* 65: 145–151 (1990).

115. Doyle, J., Biens, P., Doerenkamp, A. & Jardiné, S. Angiosperm pollen from the pre-Albian Lower Cretaceous of equatorial Africa. *Bull. Cent. Rech. Explor.-Prod. Elf-Aquitaine* **1**, 451–473 (1977).

116. Doyle, J. A. & Hotton, C. L. in *Pollen and Spores: Patterns of Diversification* (eds Blackmore, S. & Barnes, S. H.) 169–195 (Clarendon Press, Oxford, 1991).

117. von Balthazar, M., Pedersen. K. & Friis, E. *Teixeiraea lusitanica*, a new fossil flower from the Early Cretaceous of Portugal with affinities to the Ranunculales. *Plant Syst. Evol.* **255**, 55–75 (2005).

118. Knobloch, E. & Mai, D. H. Monographie der Früchte und Samen in der Kreide von Mitteleuropa. *Rozpravy Ústř. ústavu geolog, Praha* **47**, 1–219 (1986).

119. Crane, P. R., Pedersen, K. R., Friis, E. M. & Drinnan, A. N. Early Cretaceous (early to middle Albian) platanoid inflorescences associated with *Sapindopsis* leaves from the Potomac Group of Eastern North America. *Syst. Bot.* **18**, 328–344 (1993).

120. Basinger, J. F. & Dilcher, D. L. Ancient bisexual flowers. *Science* **224**, 511–513 (1984).

121. Nixon, K. C. & Crepet, W. L. Late Cretaceous fossil flowers of ericalean affinity. *Am. J. Bot.* **80**, 616–623 (1993).

122. Gandolfo, M., Nixon, K. & Crepet, W. A new fossil flower from the Turonian of New Jersey: *Dressiantha bicarpellata* gen. et sp. nov. (Capparales). *Am. J. Bot.* **85**, 964–974 (1998).

123. Góczán, F., Groot, J., Krutzsch, W. & Pacltová, B. Die Gattungen des "Stemma normapolles Pflug 1953b" (Angiospermae). *Paläont. Abh.* **2B**, 429–539 (1967).

124. Pacltová, B. Pollen grains of angiosperms in the Cenomanian Peruc Formation in Bohemia. *The Palaeobotanist* **15**, 52–54 (1966).

125. Pacltová, B. The evolution and distribution of Normapolles pollen during the Cenophytic. *Rev. Palaeobot. Palynol.* **35**, 175–208 (1981).

126. Friis, E., Pedersen, K. & Schönenberger, J. *Endressianthus*, a new normapolles producing plant genus of Fagalean affinity from the late Cretaceous of Portugal. *Int. J. Plant Sci.* **164**, S201–S223 (2003).
