# Supplementary material for: Contemporaneous radiations of fungi and plants linked to symbiosis
Source: Nat Commun. 2018 Dec 21;9:5451. doi: 10.1038/s41467-018-07849-9 (PMC6303338; doi:10.1038/s41467-018-07849-9)
Supplement: Supplementary file 2 — Description of Additional Supplementary Files [file 41467_2018_7849_MOESM2_ESM.pdf]

Description of Additional Supplementary Files

File Name: Supplementary Data 1
Description: Accession numbers for the analyzed sequence data of nine plastid genes (atpB, rbcL, psaA, psbB, rps4, matK, trnL, cpLSU, and cpSSU), including the catalytic core of the trnL group I intron for a representative sampling of green plants, and the red algal outgroup. Sequences of Asterochloris sp. (Astpho1) can be downloaded from https://genome.jgi.doe.gov/Astpho1/Astpho1.download.html.
